# Acoustic radiation characteristics of shark skin inspired surface modified plates

**DOI:** 10.1038/s41598-024-72489-7

**Published:** 2024-10-09

**Authors:** Aninda Pal, Ritwik Ghoshal

**Affiliations:** Dept of Ocean Engineering and Naval Architecture, IIT Kharagpur, Kharagpur, West Bengal 721302 India

**Keywords:** Rayleigh–Ritz method, Bio-inspiration, Plate vibration, Vibro-acoustics, Mechanical engineering, Marine biology, Bioinspired materials

## Abstract

This paper aims to evaluate the acoustic radiation characteristics of thin plates featuring a layer of small-scale biomimetic shark skin type additive surface treatment. The shark skin dermal denticles are modelled as point masses arranged in a bi-directional pattern on both the upper and lower surfaces of the plate. The governing equations are obtained through a variational approach, incorporating the Dirac Delta function in the derivation of the proposed semi-analytical model for the shark skin layer. A semi-analytical method based on the Rayleigh–Ritz formulation is utilized to analyze the vibrations of these plates with surface modification. The sound radiation characteristics are then derived from the solution of the Rayleigh integral. A comprehensive investigation is performed on the influence of surface modification on different vibro-acoustic characteristics, using a continuous structural mode and power transfer matrix-based approach. Notable observations include a reduction in peak vibro-acoustic responses with dense denticle arrangements, especially at resonance, demonstrating a direct relationship with mass ratios, i.e., the ratio of denticle mass to plate mass. The study further reveals a shift of vibro-acoustic responses towards low frequencies with an increase in mass ratios. A thorough comparative study indicates that while additive surface modifications inspired by shark skin may weaken sound radiation characteristics at resonance frequencies, a reverse effect can be observed at intermittent operational frequencies.

## Introduction

Biomimetics can be characterised as the science of mimicking some features from biological species for different engineering applications^1^. Of late, various bioinspired devices in the form of nanodevices, nanomaterials, functional surfaces, etc. have been developed from the inspiration of nature and biology^1,2^. Biomimetic shark skin has garnered significant attention from researchers. Shark skin possesses distinct tooth-like structures known as denticles or riblets, which exhibit unique attributes such as anti-biofouling properties, corrosion resistance, and drag reduction^3–5^. Marine organisms like barnacles frequently attach to ship hulls, posing potential risks such as damage or decreased vessel efficiency. The textured surface of shark skin, characterized by its denticles or small protrusions, deters such attachments. This intriguing property has led researchers to explore the replication of these microscopic dermal structures on various surfaces, especially those in marine environments. Potential applications of such surface modifications include ship hulls^6^, commercial swimwear^7,8^, interior surfaces of pipes^9,10^, and medical instruments^11,12^.

Nath et al.^13^ presented a semi-analytical model based on the Galerkin method of mean weighted residuals that emulates shark skin by incorporating a bidirectional array of denticles onto the surface of a thin plate. This model was utilized to perform a free vibration analysis of a plate featuring shark skin denticle-like surface modifications, and its results were compared with those from a full-scale finite element (FE) analysis. The simplified semi-analytical model closely aligned with the free vibration response characteristics observed in the FE analysis results. However, it is important to note that the Galerkin method requires careful selection or adaptation of basis functions to ensure a correct representation of rigid body modes. Therefore, the present study focuses on developing a semi-analytical model to study the dynamics of plates with a bidirectional array of denticle-like protuberances (biomimetic shark skin) on the surface using a Rayleigh-Ritz method that generally converges faster than the Galerkin approach using the same number of trial mode shapes^14^.

In vibration problems of rectangular plates, semi-analytical methods are generally used for solving the governing differential equation of the plate dynamics, which can be obtained using the variational approach (Hamiltonian) or from the Lagrangian (Rayleigh–Ritz)^15^ of the system. The use of the Rayleigh–Ritz formulation in vibration problems is widely explored by Meirovitch^16^, Ilanko et al.^17^ and Reddy^18^. The applicability of different types of admissible mode shape profiles in this context is studied by Meirovitch^16^ (admissible mode shapes from a set of Bessel functions, Legendre polynomials), using characteristics orthogonal polynomial obtained by Gram-Schmidt process as trial functions by Bhat^19^, and using beam characteristics functions by several researchers (Ritz^20^, Young^21^, Warburton^22^, Leissa^23^, Dickinson^24^).

Rayleigh^25^ first laid the foundation for understanding various phenomena related to the radiation of sound from a vibrating source and its interaction with different mediums/ structures. In modern engineering applications accounting vibro-acoustic characteristics such as radiated sound power, radiation efficiency, radiated sound pressure level, and directivity from a radiating sound source describes various unique characteristics of structural form. vibro-acoustics of plates is a well-defined area in sound engineering^26–30^. Acoustic radiation characteristics of a thin isotropic plate supported on all sides (SSSS) under a thermal environment were studied theoretically by Geng et al.^31^. A similar problem on composite plates was investigated by Jeyaraj et al.^32^, by employing the FE method. Chandra et al.^33^ studied the vibro-acoustic characteristics of FGM plates. Koopmann et al.^34^ explored the reduction of radiated sound power levels using added lumped masses at optimal positions (Koopmann et al.^34^, Ch. 6, p. 159), hence making the vibrating structure a weaker radiator. Li and Li^35^ examined the acoustic radiation of a plate with uniformly distributed mass patches, evaluating parameters like modal efficiency and sound power level. Significant changes in acoustic radiation were observed, particularly when the mass patch was carefully positioned at an antinode. Low et al.^36^ investigated the impact of multiple point masses of varying magnitudes on the free vibration characteristics of plates, revealing that the addition of mass modifies natural frequencies. Similarly, Kopmaz and Telli^37^ discovered that a single distributed mass influences both the natural frequencies and mode shapes. It is a widely acknowledged fact that the addition of masses to structural surfaces decreases radiated sound power, especially at localized resonance frequencies, contingent upon the positioning of the added lumped mass^34^. However, wettability modifications by resembling shark skin denticles, particularly when applied to plate surfaces, intricate bi-directional geometries emerge. Despite their minimal mass distribution relative to the base plate, these structures exhibit significantly low stiffness in practical scenarios. As a result, the dynamics of such systems are predominantly governed by the influence of the distributed point mass^13^.

The present paper primarily focuses on two aspects concerning the vibro-acoustic analysis of plates with shark skin surface modifications featuring denticles. Firstly, it aims to develop a generalized formulation for solving the free/forced vibration characteristics of a rectangular plate modelled using Kirchhoff-Love plate theory, carrying rigid spherical masses arranged in a patterned array, solved through the Rayleigh-Ritz method adaptable to various boundary conditions. Secondly, the paper addresses the estimation of sound radiation characteristics for plates with shark skin surface modifications using the solution of the Rayleigh integral. The study further explores the impact of the distributed shark skin like patterned array on the acoustic radiation of the plate, assessing parameters such as sound pressure level, equivalent radiated power, radiated sound power, and radiation efficiency. A comprehensive investigation of vibro-acoustic responses, including the directivity of the radiated sound pressure, is conducted for plates with shark skin surface modifications. This analysis takes into consideration various array spacings, boundary conditions, and base plate materials.

The outline of the remaining article is as follows: “Methodology” introduces a formulation based on the Hamiltonian of the system in the presence of surface modification. The Rayleigh-Ritz method is then applied to investigate the free and forced vibration characteristics of such a system. Additionally, in “Methodology”, the formulation to compute sound radiation characteristics from the Rayleigh integral, including equivalent radiated power, radiated sound power, and sound pressure level, is discussed. In “Validation”, a validation study is presented for both the vibration/structural and acoustic models, utilizing the formulation introduced in this study. The section further conducts a parametric study, considering various base plate materials, boundary conditions, and denticle spacings, with corresponding results and discussions that include several observations. Finally, the conclusions are drawn in “Conclusions” based on this comprehensive study.

## Methodology

The study investigates the vibro-acoustics response of a plate-type structure featuring attached shark skin denticles designed to modify its wettability. Under the action of loading, the plate vibrates in the air, and pressure fluctuations within the adjacent air region are induced by the velocity of the plate. These small-scale pressure fluctuations are radiated outward from the source. The radiation characteristics of the wettability-modified plates are further investigated.

### Vibrations of plates modified with wettability features inspired from shark skin

**Figure 1:**
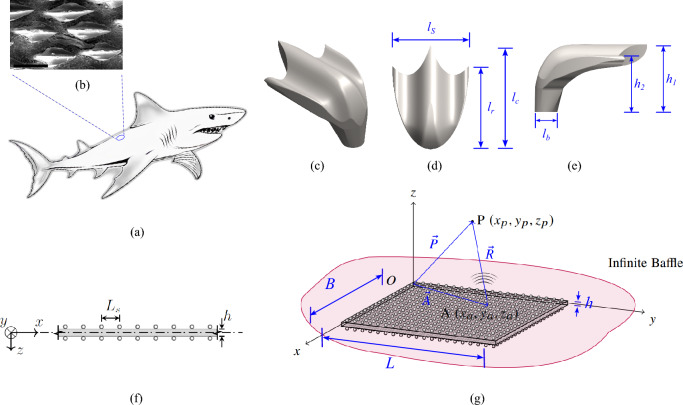
Schematic representation depicting a shark (**a**) with shark skin (**b**), specifically referencing the bigeye thresher shark (*Alopias superciliosus*) as illustrated in^38^. Additionally, (**c**) showcases modified three-dimensional geometry, while (**d,e**) illustrate various denticle parameters in compliance with Nath et al.^13^. A schematic representation of a plate with biomimetic shark skin wettability is provided for simplified semi-analytical modelling in (**f**)  and (**g**). (**f**) Showcases a cross-sectional view of the idealized plate in two dimensions, with (**g**) schematic description of sound radiation from a vibrating plate with shark skin denticle-like surface modifications in an infinite baffle. Shark skin denticles are idealized as rigid point masses on both the top and bottom surfaces of the plate. $L_s$ denotes the distance between two consecutive denticles in the *x* and *y* directions. The middle plane is situated at $z = 0$. An arbitrary point $\overrightarrow{P}(x_p, y_p, z_p)$ is positioned at different distances from these radiators $\overrightarrow{A}(x_a, y_a, z_a)$.

In the present formulation, a homogeneous, isotropic uniform rectangular plate based on Kirchhoff-Love plate theory is considered. The proposed model treats the denticles as discrete point masses distributed over the plate in a patterned manner on both sides of the plate. These denticles are assumed to be rigid and do not contribute to additional potential energy under vibration. Figure 1 depicts a schematic of the plate with shark skin surface modification. Its dimensions are characterized by its length *L*, width *B*, and thickness *h*. The plate is presumed to span in both the *x* and *y* directions, while its thickness is along the *z* direction, with boundaries of the plate domain defined by the top surface at $z=-h/2$ and the bottom surface at $z=h/2$. The undeformed middle plane of the plate is denoted as $\Omega$. The overall domain of the plate is characterized by the tensor product $\Omega \times (-h/2, h/2)$.

#### Strain–displacement relations

The implication of the Kirchhoff hypothesis^18^ results in the following form of the displacement field for time (*t*) dependent deformations :

1 $$\begin{aligned} \! \begin{aligned} u(x,y,z,t)&= u_0(x,y,t)- z\frac{\partial w_0(x,y,t)}{\partial x}, \\ v(x,y,z,t)&= v_0(x,y,t)- z\frac{\partial w_0(x,y,t)}{\partial y}, \\ \text {and} ~ w(x,y,z,t)&= w_0(x,y,t). \end{aligned} \end{aligned}$$

Here $$\mathbf {u_0}=\begin{Bmatrix} u_0&v_0&w_0 \end{Bmatrix}^T$$ is the displacement field of the mid-plane of the plate, and $$\textbf{u}=\begin{Bmatrix} u&v&w \end{Bmatrix}^T$$ represents the displacement field of the plate domain (where $x:x\in [0,L]$ and $y:y\in [0,B]$).

For cases involving small strains and moderate rotations, the strain-displacement relations can be described by simplifying the Green-Lagrange strain tensor^18^ as

2 $$\begin{aligned} \begin{Bmatrix} \epsilon _{xx} \\ \epsilon _{yy} \\ \epsilon _{xy} \end{Bmatrix}= \frac{1}{2} \begin{Bmatrix} 2u_{0,x}+w_{0,x}^2 \\ 2v_{0,y}+w_{0,y}^2 \\ u_{0,y}+v_{0,x}+w_{0,x}w_{0,y} \end{Bmatrix} -z \begin{Bmatrix} {w_{0,xx}} \\ {w_{0,yy}} \\ {2w_{0,xy}} \end{Bmatrix} \end{aligned}$$

Here, {$\epsilon _{xx}$ $\epsilon _{yy}$ $\epsilon _{xy}\}^T$ represents the strain tensor (only keeping the non-zero terms) of an infinitesimal plate element undergoing transverse vibrations following previous approximations.

#### Equations of motion of a plate with surface modification

The governing dynamics of the vibrating plate system with surface modification are formulated using Hamilton’s principle^18^,

3 $$\begin{aligned} \int _{t_1}^{t_2} (\delta \mathcal {U} +\delta \mathcal {V}-\delta \mathcal {K} )dt=0, \end{aligned}$$

where $\mathcal {U}$ is strain energy, $\mathcal {V}$ is the work done due to the external source, and $\mathcal {K}$ represent the kinetic energy. $t_1$ and $t_2$ represent the initial and final times, respectively. The symbol ‘$\delta$’ refers to the variation and when employed in the precursor, it signifies the variation of the subsequent quantity.

In the presented semi-analytical model, it is assumed that denticles do not impart any bending stiffness; rather, their sole contribution lies in inertia. This is based on previous studies that utilised high-fidelity 3D FE simulations, accurately modelling the shape of the denticles^13^. These studies indicate that the addition of mass substantially enhances kinetic energy during plate vibration, with minimal impact on the potential energy. Consequently, the variation in strain energy ($\delta \mathcal {U}$) for a wettability-modified plate remains identical to that of a plate without any surface modification and can be expressed as^18^

4 $$\begin{aligned} \begin{aligned} \delta \mathcal {U} = \int _{\Omega }\int _{-h/2}^{h/2} (\sigma _{xx}\delta \epsilon _{xx}+\sigma _{yy}\delta \epsilon _{yy}+\sigma _{xy}\delta \epsilon _{xy}) \,dz\,d\Omega . \end{aligned} \end{aligned}$$

It is important to highlight that the incorporation of denticles ($m_o$) on both the top and bottom of the plate results in additional virtual kinetic energy, a factor duly considered in the calculation of kinetic energy. The expression for total kinetic energy ($\delta \mathcal {K}$) becomes

5 $$\begin{aligned} \delta \mathcal {K} =\delta \mathcal {K}_0+\delta \mathcal {K}_a. \end{aligned}$$

$\delta K_a$ is the additional kinetic energy contribution due to the addition of denticles. $\delta \mathcal {K}_0$ is the kinetic energy ($\delta \mathcal {K}$) for a plate without surface modification and can be expressed as^18^

6 $$\begin{aligned} \delta \mathcal {K}_0 = \int _{\Omega }\int _{-h/2}^{h/2} \rho _p(\dot{u}\delta \dot{u}+\dot{v}\delta \dot{v}+\dot{w}\delta \dot{w}) \,dz\,d\Omega . \end{aligned}$$

Here, $\rho _p$ is the areal density of the plate material. $(\dot{\vphantom{o}})$ and $(\ddot{\vphantom{o}})$ represent the first and second-order derivative with respect to the time *t*. In Eq. (6), the components of virtual kinetic energy arising from the three velocities, i.e., $\dot{u}\delta \dot{u}$, $\dot{v}\delta \dot{v}$, and $\dot{w}\delta \dot{w}$, can be derived by substituting the displacement field defined in Eq. (1) as follows:

7 $$\begin{aligned} \begin{aligned} \dot{u}\delta \dot{u}&= u_{0,t}\delta u_{0,t}-z(w_{0,xt}\delta u_{0,t}+u_{0,t}\delta w_{0,xt})+z^2w_{0,xt}\delta w_{0,xt}, \\ \dot{v}\delta \dot{v}&= v_{0,t}\delta v_{0,t}-z(w_{0,yt}\delta v_{0,t}+v_{0,t}\delta w_{0,yt})+z^2w_{0,yt}\delta w_{0,yt}, \\ \dot{w}\delta \dot{w}&= w_{0,t}\delta w_{0,t}. \end{aligned} \end{aligned}$$

In the case of a plate without surface modification (in the absence of $\delta \mathcal {K}_a$), the governing plate dynamics can be found using Eqs. (3)–(7), as discussed in a wide range of literature^17,18^. If the rotary inertia of the plate mass is neglected due to the thin plate assumption, then for a differential element without any excitation force, the corresponding differential form $m\ddot{w} + D\nabla ^4w = 0$ is found by considering only the $\delta \mathcal {U}$ and $\delta \mathcal {K}$ terms in Eq. (3), following standard steps^18^.

The introduction of denticles ($m_o$) on both the top and bottom of the plate results in an increase in the kinetic energy of the plate during vibration. Using the Dirac delta operator ($\varvec{\delta }$), the mass distribution ($m_d$) is modeled as

8 $$\begin{aligned} m_d=m_o\varvec{\delta }(x-x_i)\varvec{\delta }(y-y_j). \end{aligned}$$

where ($x_i,y_j$) represents the position of the added mass. Subsequently, the additional kinetic energy contribution due to the added denticles ($\delta K_a$) turns out to be

9 $$\begin{aligned} \begin{aligned} \delta \mathcal {K}_a =\int _{\Omega }\int _{-h/2}^{h/2} m_d(\dot{u}\delta \dot{u}+\dot{v}\delta \dot{v}+\dot{w}\delta \dot{w})\mid _{z= -\frac{h}{2}} \,dz\,d\Omega .+ \\ \int _{\Omega }\int _{-h/2}^{h/2} m_d(\dot{u}\delta \dot{u}+\dot{v}\delta \dot{v}+\dot{w}\delta \dot{w})\mid _{z= \frac{h}{2}} \,dz\,d\Omega . \end{aligned} \end{aligned}$$

It is important to note that since the surface modifications are done on both sides of the plate, the additional kinetic energy contribution due to the added denticles on both surfaces must be considered in the governing equation for plate dynamics. In the present formulation, the contribution from added denticles on both surfaces is treated separately. The additional kinetic energy due to an array of added denticle mass on the top surface of the plate obtained as

10 $$\begin{aligned} \begin{aligned}{}&\int _{\Omega }\int _{-h/2}^{h/2} m_d(\dot{u}\delta \dot{u}+\dot{v}\delta \dot{v}+\dot{w}\delta \dot{w})\mid _{z= -\frac{h}{2}} \,dz\,d\Omega \\&=\sum \int _{\Omega }\int _{-h/2}^{h/2} m_d \Biggl [ \begin{Bmatrix} u_{0,t}\delta u_{0,t} \\ v_{0,t}\delta v_{0,t} \\ w_{0,t}\delta w_{0,t} \end{Bmatrix} -z \begin{Bmatrix} (w_{0,xt}\delta u_{0,t}+u_{0,t}\delta w_{0,xt}) \\ (w_{0,yt}\delta v_{0,t}+v_{0,t}\delta w_{0,yt}) \\ 0 \end{Bmatrix} +z^2 \begin{Bmatrix} w_{0,xt}\delta w_{0,xt} \\ w_{0,yt}\delta w_{0,yt} \\ 0 \end{Bmatrix} \Biggr ]\mid _{z= -\frac{h}{2}} \,dz\,d\Omega ,\\&=\sum \int _{\Omega }\int _{-h/2}^{h/2} m_d \Biggl [ \begin{Bmatrix} u_{0,t}\delta u_{0,t} \\ v_{0,t}\delta v_{0,t} \\ w_{0,t}\delta w_{0,t} \end{Bmatrix} +\frac{h}{2} \begin{Bmatrix} (w_{0,xt}\delta u_{0,t}+u_{0,t}\delta w_{0,xt}) \\ (w_{0,yt}\delta v_{0,t}+v_{0,t}\delta w_{0,yt}) \\ 0 \end{Bmatrix} +\frac{h^2}{4} \begin{Bmatrix} w_{0,xt}\delta w_{0,xt} \\ w_{0,yt}\delta w_{0,yt} \\ 0 \end{Bmatrix} \Biggr ] \,dz\,d\Omega . \end{aligned} \end{aligned}$$

Here, $\dot{u}\delta \dot{u}$, $\dot{v}\delta \dot{v}$, and $\dot{w}\delta \dot{w}$, are taken from the Eq. (7). Similarly, the contribution due to the added denticle mass on the bottom surface gives rise to the additional kinetic energy as

11 $$\begin{aligned} \begin{aligned}{}&\int _{\Omega }\int _{-h/2}^{h/2} m_d(\dot{u}\delta \dot{u}+\dot{v}\delta \dot{v}+\dot{w}\delta \dot{w})\mid _{z= \frac{h}{2}} \,dz\,d\Omega \\&=\sum \int _{\Omega }\int _{-h/2}^{h/2} m_d \Biggl [ \begin{Bmatrix} u_{0,t}\delta u_{0,t} \\ v_{0,t}\delta v_{0,t} \\ w_{0,t}\delta w_{0,t} \end{Bmatrix} -\frac{h}{2} \begin{Bmatrix} (w_{0,xt}\delta u_{0,t}+u_{0,t}\delta w_{0,xt}) \\ (w_{0,yt}\delta v_{0,t}+v_{0,t}\delta w_{0,yt}) \\ 0 \end{Bmatrix} +\frac{h^2}{4} \begin{Bmatrix} w_{0,xt}\delta w_{0,xt} \\ w_{0,yt}\delta w_{0,yt} \\ 0 \end{Bmatrix} \Biggr ] \,dz\,d\Omega . \end{aligned} \end{aligned}$$

Therefore, the total kinetic energy contribution from the denticles on both surfaces can be obtained by summing up Eqs. (10) and (11). This yields an additional term, $2 m_d(\ddot{w} + \frac{h^2}{4}\nabla ^2 w)$, in the corresponding differential form. Here, the term containing $h$ accounts for the additional rotary inertia of the added denticles. The influence of the offset of the denticles from the mid-plane is evident from the expression. Subsequently, for a thin elastic isotropic plate^18^, the equation of motion for the plate dynamics with surface modifications on the top and bottom surfaces can be expressed as follows:

12 $$\begin{aligned} \text {Free vibration:}~ 2 m_d(\ddot{w}+\frac{h^2}{4}\nabla ^2 w)+m\ddot{w}+D\nabla ^4w=0. \end{aligned}$$

13 $$\begin{aligned} \text {Force vibration:}~ 2 m_d(\ddot{w}+\frac{h^2}{4}\nabla ^2 w)+m\ddot{w}+D\nabla ^4w+P_d=P_E. \end{aligned}$$

Here, $\nabla ^2$ is the Laplace operator and $\nabla ^4$ is the Bi-harmonic operator. $D= \frac{E h^3}{12 (1 -\nu ^2)}$ stands for the bending stiffness of the plate. *E* is the Young’s modulus, and $\nu$ is the Poisson’s ratio of the plate material. $P_d$ and $P_E$ represent the damping and external forces in the transverse direction. The Eqs. (12) and (13) represent the strong form of the governing equation of motion for the plate system vibrating in the air. However, it is important to note that if the fluid medium is water, the added hydrodynamic mass plays a crucial role in determining the free and forced vibrational characteristics of the plate. In such cases, the hydrodynamic effect results in cross-coupling between the modes, leading to non-orthogonal free vibration mode shapes^27^. As the density of the fluid decreases, the added mass effect gradually diminishes, and in the case of plate vibration in the air, it can be simplified to plate vibration in a vacuum.

### Solution method for plate vibration with wettability modification

This section formulates the governing dynamic response of plates with shark skin denticle-like wettability modifications. The corresponding surface modifications are represented as a plate with a two-dimensional array of distributed mass. The governing differential equation on a plate domain $\Omega \subset {\mathbb R}^{d}$ with a given boundary condition, the $L^{2}$-inner product can be used to derive the weak formulation as

14 $$\begin{aligned} \langle R, W_a \rangle = 0, \end{aligned}$$

where *R* is the equation residual and $W_a$ represents a set of admissible functions. Following this, the strong form of the governing differential equation (Eq. 12) can be transformed into its corresponding weak form, resulting in an equivalent energy formulation for plate dynamics. Consequently, the total energy stored in the system ($\Pi$) can be defined as the summation of kinetic energy ($\mathcal {K}$) and potential energy ($\mathcal {U}$),

15 $$\begin{aligned} \Pi = \mathcal {K}+\mathcal {U}= \mathcal {K}_0+\mathcal {K}_a+\mathcal {U}. \end{aligned}$$

Here, $\mathcal {U}$ represents the strain energy associated with undamped free vibration in a linear elastic plate,

16 $$\begin{aligned} \mathcal {U}= \int _{\Omega }D\Bigl \{w_{,xx}^2+w_{,yy}^2+2(1-\nu )w_{,xy}^2+2 \nu w_{,xx} w_{,yy} \Bigl \} \,d\Omega , \end{aligned}$$

The kinetic energy of the bare plate ($\mathcal {K}_{0}$), i.e., without the denticle mass arrangement, is obtained as

17 $$\begin{aligned} \mathcal {K}_{0} = - \rho h \omega ^2 \int _{\Omega } w^2 \, d\Omega , \end{aligned}$$

and the additional kinetic energy ($\mathcal {K}_{a}$) due to the plate vibration with the arrays of denticle mass arrangement becomes

18 $$\begin{aligned} \mathcal {K}_{a}=-2m_d \omega ^2 \Bigl \{\int _{\Omega } w^2 \, d\Omega -\frac{h^2}{4}\int _{\Omega } (w_{,xx}^2+w_{,yy}^2) \, d\Omega \Bigl \}. \end{aligned}$$

Now, using the method of separation of variables, the transverse displacement ($w$) can be represented as

19 $$\begin{aligned} w(x,y,t) = S(x,y)T(t)= S(x,y) e^{i\omega t}, \end{aligned}$$

where *S*(*x*, *y*) is the spatial and *T*(*t*) is the temporal component of the transverse displacement, respectively.

### Semi-analytical solution of plate vibration

In this section, a semi-analytical Rayleigh-Ritz (RR) energy approach is utilized to determine the configuration of modal distribution at which the total energy attains its local minimum. Initially, the trial plate mode shapes are assumed, and the weighted superposition of suitable mode shapes along the $x$ and $y$ directions is executed, i.e., $S(x,y) = V X(x)Y(y) = V_{ij} X_{i}(x) Y_{j}(y) = V_{ij}\mathcal {W}(x,y)$. Here, $V = V_{ij}$ denotes the modal coefficients, whereas $\mathcal {W} = \mathcal {W}_{ij}$ signifies a set of trial functions. The trial function $\mathcal {W}_{ij}$ is chosen^39,40^ in such a way that:

1. The trial mode shapes $X(x)$ and $Y(y)$ are chosen from the same family and must form a complete set of the first $N$ members, with no modes missing.
2. The trial mode shapes must be linearly independent.
3. The trial mode shapes should satisfy the geometric boundary conditions.
4. The trial mode shapes must have derivatives at least up to half of the order of the partial differential equation.

The assumed trial mode shapes for the present study involve a weighted combination of the mode shapes of Euler-Bernoulli (EB) beams in both the $x$ and $y$ directions, which satisfy the above requirements. The mode shapes of an EB beam that spans in the $x$ direction can be represented as^28^:

20 $$\begin{aligned} \phi (x) = A_1 \sin (gx) + A_2 \cos (gx) + A_3 \sinh (gx) + A_4 \cosh (gx), \end{aligned}$$

where $A_1$, $A_2$, $A_3$, and $A_4$ are arbitrary constants and can be determined through the boundary conditions, as detailed in Table 1. The parameter $g$ can be obtained from the geometric and material properties of the beams.

Addressing structural vibration problems with various boundary conditions analytically often proves challenging because of the rigid body modes. In the set of trial mode shapes, suitable pre-positioning of the trial mode shapes must be done according to Table 2 for suitable boundary conditions. This investigation conducts vibro-acoustic analysis for eight distinct plate boundary conditions: SSSS, SCSC, CCFF, CCCC, CFCF, CFSF, CFFF, and CSCG.

#### Free vibration

**Table 1:** Combining beam boundary conditions with conditions for $\phi$ and its derivatives.

Boundary conditions	1	2
Simply supported (S)	$\phi =0$	$\phi ''=0$
Clamped / Fixed (C)	$\phi =0$	$\phi '=0$
Gliding / Sliding (G)	$\phi '=0$	$\phi '''=0$
Free (F)	$\phi ''=0$	$\phi '''=0$
$(~)^{'}$, $(~)^{''}$, and $(~)^{'''}$ represent the first. second and third order derivative with respect to the independent variable respectively

**Table 2:** Rigid body modes (For beam spanning in *x* direction).

Boundary conditions	Number of rigid body modes	Rigid body mode shape		
		Mode	1	2
FF	2		1	$1-2\frac{x}{L}$
GF	1		1	_
GG	1		1	_

In the context of the Rayleigh–Ritz method for free vibration analysis of plates with wettability modifications, the objective is to determine the natural frequencies and corresponding mode shapes. This involves minimizing the total energy ($\Pi$) with respect to the unknown modal amplitude coefficients $V_{ij}$.

21 $$\begin{aligned} \frac{\partial \Pi }{\partial V_{ij}} = 0, i=1,2,3,..., m; j=1,2,3,..., n; \end{aligned}$$

and this leads to the formulation of an eigenvalue problem^40^. The unknown coefficients $V_{ij}$ can be obtained by solving the eigenvalue problem in the matrix form of

22 $$\begin{aligned} \begin{aligned} \Big [- \omega ^2({\textbf {M}}^{a}+{\textbf {M}})+ {\textbf {K}}\Big ]\{V\}= 0, \end{aligned} \end{aligned}$$

here eigenvalues represent the natural frequencies ($\omega$) of the plate and the eigenvector ($\{V\}$) gives the mode-shapes related to corresponding natural frequencies. Here, (${\textbf {M}}$) represents the mass matrix, (${\textbf {M}}^{a}$) denotes the denticle mass matrix, and (${\textbf {K}}$) stands for the stiffness matrix are obtained as follows:

23 $$  \begin{aligned} \text {Mass matrix} && M_{mn ij} = \frac{\partial \mathcal {K}_{0}}{\partial V_{mn}}\frac{\delta _{ij mn}}{V_{ij}} \end{aligned}$$

24 $$\begin{aligned} \text {Denticle mass matrix} && M^a_{mn ij} = \frac{\partial \mathcal {K}_{a}}{\partial V_{mn}}\frac{\delta _{ij mn}}{V_{ij}} \end{aligned}$$

25 $$\begin{aligned} \text {Stiffness matrix} && K_{mn ij} = \frac{\partial \mathcal {U}}{\partial V_{mn}}\frac{\delta _{ij mn}}{V_{ij}} \end{aligned}$$

Here $\delta _{ijmn}$ is analogous to $\delta _{pq}$ and it represents the Kronecker delta and (*p*, *q*) : (*p*, *q*) $\in$ { (1, 1), (1, 2), (2, 1), ...}.

#### Forced vibration

The general equation of motion for the forced vibration of a plate with damping ratio $\xi$ can be solved using the weighted residual method and expressed in modal coordinates $\zeta (t)$. The natural frequency ($\varvec{\omega }$) and modal coefficients (${\textbf {V}}$), determined from the free vibration analysis, are employed in the forced vibration analysis. Accordingly, the governing equation takes the following form :

26 $$\begin{aligned} \begin{aligned} ({\textbf {GM}}^{a}+{\textbf {GM}})\{\ddot{\zeta }(t)\}+ {\textbf {GK}}\{\zeta (t)\}+ {\textbf {GD}}\{\dot{\zeta }(t)\}= {\textbf {GF}}(t), \end{aligned} \end{aligned}$$

where **GD** is the generalized damping and can be expressed as

27 $$\begin{aligned} \begin{aligned} {\textbf {GD}}= 2\xi [\varvec{\omega }]({\textbf {GM}}^{a}+{\textbf {GM}}). \end{aligned} \end{aligned}$$

Here, ${\textbf {GM}}=[{\textbf {V}}]^T {\textbf {M}} [{\textbf {V}}]$, ${\textbf {GM}}^{a}=[{\textbf {V}}]^T {\textbf {M}}^{a}[{\textbf {V}}]$, ${\textbf {GK}}=[{\textbf {V}}]^T{\textbf {K}}[{\textbf {V}}]$, and ${\textbf {GF}}=[{\textbf {V}}]^T{\textbf {F}}$ correspond to the generalized mass, generalized denticle mass, generalized stiffness, and generalized force matrix, respectively.

The expression for the generalized force vector is given as

28 $$\begin{aligned} F_{mn} = \iint \limits _\Omega \mathcal {P}(x,y,t).\mathcal {W}_{mn}\,dx\,dy. \end{aligned}$$

When utilizing a generalized coordinate $\zeta (t)$, the dynamic system transforms into a set of $\mathcal {N}$ second-order ordinary differential equations with time as the independent variable. Here, $\mathcal {N}$ signifies the number of modes considered in the study.

In the case of harmonic analysis, a point load with frequency $\omega _f$ is applied at the top of the plate at a specific point. This frequency range is optimally chosen to reflect the operational frequency range under which the model has to perform. The mathematical representation of the unit point load involves the utilization of a Dirac Delta function. In the case of harmonic forcing, it is expressed as a complex exponential function,

29 $$\begin{aligned} \mathcal {P}(x,y,t) = \mathscr {P} \delta (x-x_f)\delta (y-y_f) e^{j\omega _f t}, \end{aligned}$$

where the coordinate ($x_f$, $y_f$) indicates the specific point where the load is applied, and $\omega _f$ represents the angular frequency. $\mathscr {P}$ refers to the magnitude of the applied load.

In the scenario of predominant hysteresis damping characterized by the loss factor ($\eta$), the complex displacement time history ($\tilde{\zeta }$) in the generalized coordinate can be determined for the complex loading (Eq. 29). $\tilde{\zeta }$ is a function of forcing frequency ($\omega _f$) and time (*t*) for the *mn*-th generalized co-ordinate and it can be expressed as

30 $$\begin{aligned} {\tilde{\zeta }}_{mn}(\omega_{f,t}) = \frac{GF_{mn}}{GK_{mn}\left[ 1-(\frac{\omega _f}{\omega _{mn}})^2+j\eta \right] }, \end{aligned}$$

The steady-state complex displacement response of the plate as a function of space and time ($\tilde{w}(x,y,t)$) can be expressed as

31 $$\begin{aligned} \tilde{w}(x,y,\omega _f,t)=\{\tilde{\zeta }(\omega _f,t)\}^T [{\textbf {V}}]^T \{S(x,y)\}=\{\tilde{\zeta }(\omega _f,t)\}^T \varvec{\phi }(x,y). \end{aligned}$$

Here $[{\textbf {V}}]^T \{S(x,y)\}=\varvec{\phi }(x,y)$ is the set of the normalised mode shapes, and $\tilde{\zeta }(\omega _f,t)$ is the complex modal displacement. The steady-state complex modal velocity ($\tilde{v}$) can be obtained from the time derivative of the complex displacement quantity,

32 $$\begin{aligned} \tilde{v}(x,y,\omega _f,t)=\{\dot{\tilde{\zeta }}(\omega _f,t)\}^T \varvec{\phi }(x,y). \end{aligned}$$

Consequently, the corresponding acceleration response can be derived from the second-order time derivative of Eq. (31).

The driving point velocity under the excitation point ($x_f, y_f$) can be determined by extracting the real part of the velocity amplitude, expressed as $v_d=\Re (\tilde{v}(x_f, y_f, \omega _f, t))$. where $\Re$ signifies the real part of the expression. In this current investigation, the primary objective of conducting forced vibration analysis is to examine the radiation characteristics of plates featuring modifications in wettability resembling shark skin.

### Vibro-acoustic response of bio-inspired plate using the structural modes

Sound radiation characteristics of plates in multi-mode transverse vibrations can be expressed in terms of structural vibrational modes^27^. In the analysis presented, the plate under consideration is assumed to be placed in an infinite baffle, as illustrated in Fig. 1g. A unit harmonically varying load ($\mathscr {P}=1$ N) is exerted at a specific point on the model and various vibro-acoustics response parameters across a predefined frequency range are studied.

#### Radiated sound pressure

Acoustic radiation from a vibrating plate can be obtained using the Rayleigh integral, through a superposition of multi-modal responses. The surface of the vibrating plate acts as an infinite array of radiators. The formula for pressure fluctuation ($\tilde{p}$) at a point ($\overrightarrow{P}(x_p, y_p, z_p)$ in Fig. 1g), positioned at different distances from these radiators ($\overrightarrow{A}(x_a, y_a, z_a)$ in Fig. 1g), can be expressed as^27^

33 $$\begin{aligned} \begin{aligned} \tilde{p}(x_p,y_p,z_p,\omega _f,t)=\frac{j\omega _f\rho _a}{2\pi }\int _{0}^{L}\int _{0}^{B}\tilde{v}(x_a,y_a,\omega _f,t) \frac{e^{-jkR}}{R}\,dy_a\,dx_a, \end{aligned} \end{aligned}$$

where *R* is the magnitude of the vector defining the difference between the point of interest within the acoustic field $\overrightarrow{P}(x_p,y_p,z_p)$ and the position of the elementary radiator on the plate $\overrightarrow{A}(x_a,y_a,0)$, i.e., $|\overrightarrow{R}|=|\overrightarrow{P}-\overrightarrow{A}|=\sqrt{(x_p-x_a)^2+(y_p-y_a)^2+z_p^2}$ (see Fig. 1)g. $\rho _a$ represents the density of the air medium. *k* is the wave number, calculated as $k = \omega _f / c_a$, where $\omega _f$ is the forcing frequency and $c_a$ is the sound speed. Subsequently, the radiated sound pressure is expressed as the Sound Pressure Level (SPL) in the decibel scale using the equation^41^

34 $$\begin{aligned} p_{dB}= 20\log _{10} \frac{p_{rms}}{p_{ref}}, \end{aligned}$$

The reference sound pressure $p_{ref}$ is considered to be $20\times 10^{-6}$ Pa.

Here, the root mean square pressure $p_{\text {rms}}$ can be obtained only after the pressure response reaches a steady state at time $T_0$. It can be calculated as^41^

35 $$\begin{aligned} p_{\text {rms}} = \biggl [\frac{1}{T}\int _{T_0}^{T_0+T} \tilde{p}^* \tilde{p} \,dt\biggr ]^\frac{1}{2}, \end{aligned}$$

where the superscript ‘$^*$’ denotes the complex conjugate.

#### Radiated sound power level

The time-averaged sound power radiation ($W(\omega _f)$) can be obtained by integrating (real part) of the acoustic intensity, i.e., the product of the surface sound pressure $\tilde{p}(x_p,y_p,0,\omega _f,t)$, and the transverse velocity of the panel $\tilde{v}(x_a,y_a,\omega _f,t)$ over the entire surface of a plate ($\Omega$)^27^,

36 $$\begin{aligned} W(\omega _f) =\ \frac{1}{2}\Re \Bigl \{\int _{0}^{L}\int _{0}^{B}\tilde{v}(x_p,y_p,\omega _f,t)^{*}\tilde{p}(x_p,y_p,0,\omega _f,t)\,dy_p\,dx_p\Bigr \}, \end{aligned}$$

Substituting expression of pressure fluctuation ($\tilde{p}$) from Eq. (33) into Eq. (36) yields

37 $$\begin{aligned} \begin{aligned} W(\omega _f) =\frac{1}{2}\Re \Bigl \{\frac{j\omega_f \rho _a}{2\pi }\int _{0}^{L}\int _{0}^{B}\int _{0}^{L}\int _{0}^{B}\tilde{v}(x_p,y_p,\omega _f,t)^{*} \tilde{v}(x_a,y_a,\omega _f,t)\frac{e^{-jkR}}{R}\,dy_p\,dx_p\,dy_a\,dx_a\Bigr \} \end{aligned} \end{aligned}$$

Using complex modal velocity ($\tilde{v}$) of Eq.. 32, Eq. 37 can be rewritten as

38 $$\begin{aligned} \begin{aligned} W(\omega _f)= \frac{1}{2}\Re \Bigl \{\frac{j\omega _f\rho _a}{2\pi }\int _{0}^{L}\int _{0}^{B}\int _{0}^{L}\int _{0}^{B}\{\dot{\tilde{\zeta }}(\omega _f,t)\}^H \varvec{\phi }(x,y)^T\frac{e^{-jkR}}{R} \varvec{\phi }(x,y)\{\dot{\tilde{\zeta }}(\omega _f,t)\}\,dy_p\,dx_p\,dy_a\,dx_a\Bigr \}. \end{aligned} \end{aligned}$$

where *H* denotes a Hermitian transpose. It is important to note that the surface of the plate is designated as the origin of acoustic radiation. Consequently, the variable *R*, denoted as $R=\sqrt{(x_p-x_a)^2+(y_p-y_a)^2}$, signifies the distance between the point of interest within the acoustic field $\overrightarrow{P}(x_p, y_p, 0)$ and the location of the elementary radiator on the plate $\overrightarrow{A}(x_a, y_a, 0)$ (see Fig. 1g).

It is noteworthy that only the real component of the integral expression in Eq. (38) will contribute to the acoustic power output of the structure. Consequently, Eq. (38) can be simplified as

39 $$\begin{aligned} \begin{aligned} W(\omega _f) = \frac{\omega_f \rho _a}{4\pi }\{\dot{\tilde{\zeta }}(\omega _f,t)\}^H\Bigl \{\int _{0}^{L}\int _{0}^{B}\int _{0}^{L}\int _{0}^{B} \varvec{\phi }(x,y)^T \frac{sin kR}{R} \varvec{\phi }(x,y)\,dy_p\,dx_p\,dy_a\,dx_a\Bigr \}\{\dot{\tilde{\zeta }}(\omega _f,t)\}. \end{aligned} \end{aligned}$$

The Eq. (39) can be expressed in a more compact form as

40 $$\begin{aligned} W(\omega _f) = \dot{\tilde{\zeta }}(\omega _f,t)^H \mathbb {A}(\omega _f) \dot{\tilde{\zeta }}(\omega _f,t). \end{aligned}$$

Here, $\mathbb {A}(\omega _f)$ is a symmetric and positive definite matrix, often referred to as the ‘power transfer matrix’, which can be expressed as^27^

41 $$\begin{aligned} \begin{aligned} \bigl [ \mathbb {A}(\omega _f)\bigr ]=\int _{0}^{L}\int _{0}^{B}\int _{0}^{L}\int _{0}^{B} \varvec{\phi }(x,y)^T\frac{sin kR}{R} \varvec{\phi }(x,y) \,dy_p\,dx_p\,dy_a\,dx_a. \end{aligned} \end{aligned}$$

Note that to determine $\mathbb {A}(\omega _f)$, it is essential to evaluate the quadruple integral expression presented in Eq. (41). In this particular problem, the integrand does not exhibit any poles, allowing the utilization of Gaussian quadrature for the matrix evaluation.

Consequently, Eq. (40) can be expressed in terms of sound power level in decibels as^26^

42 $$\begin{aligned} W_{dB} = 10\log _{10} \frac{W}{W_{ref}}, \end{aligned}$$

where reference sound power $W_{ref}$ is considered as $1\times 10^{-12}$ watts.

#### Equivalent sound power level

Equivalent radiated power (ERP)^42^ is a simplified power radiation model in which radiated power is expressed as a function of the spatial average of mean square velocity over the plate surface. For a complex harmonic loading, the ERP ($W_{ERP} (\omega _f)$) is defined as

43 $$\begin{aligned} W_{ERP} (\omega _f) = \rho _ac_aLB<\bar{V}^2_{n}>. \end{aligned}$$

Here, $<\bar{V}^2_{n}>$ is the space-averaged mean square velocity and it is determined as

44 $$\begin{aligned} <\bar{V}^2_{n}> = \frac{1}{2LB}\int _{\Omega } \tilde{v}(x,y,\omega _f,t)^T \tilde{v}(x,y,\omega _f,t) d\Omega . \end{aligned}$$

Consequently, ERP in decibels can be obtained through the expression

45 $$\begin{aligned} ERP_{dB} = 10\log _{10} \frac{W_{ERP}}{W_{ref}}, \end{aligned}$$

where reference sound power $W_{ref}$ is considered as $1\times 10^{-12}$ watts.

#### Radiation efficiency

The radiation efficiency ($\sigma$) of a structure is a measure of the radiation capability of a vibrating object. The total radiation efficiency of the plate turns out to be the ratio of the time-averaged sound power radiation ($W (\omega _f)$) to the equivalent radiated power ($W_{ERP} (\omega _f)$). This relationship can be expressed mathematically as^27^:

46 $$\begin{aligned} \sigma (\omega _f) = \frac{W (\omega _f)}{W_{ERP} (\omega _f)}. \end{aligned}$$

Here, $W (\omega _f)$ can be obtained from Eq. (40) and $W_{ERP} (\omega _f)$ can be calculated using the definition in Eq. (44). This ratio provides insight into how efficiently the vibrating object converts mechanical energy into radiated acoustic power, offering a comprehensive measure of its radiation performance.

## Results and discussions

The current study focuses on evaluating the sound radiation characteristics of plates undergoing multi-mode transverse vibrations. Specifically, parameters such as radiated sound pressure, radiated sound power level, and radiation efficiency is computed for plates modified with shark skin wettability. Therefore, at first, the proposed mathematical formulation is validated through a comparison of the frequency response and vibro-acoustic characteristics of a bare plate with those found in the existing literature. A comparative analysis is then conducted by comparing these characteristics with those of the unmodified or bare plates. The influence of surface modification resembling shark skin on radiated sound pressure, equivalent radiated power, sound power level, and directivity is extensively examined across eight distinct boundary conditions, three varying shark skin denticle spacings, and three diverse materials for the base plate. The goal is to assess and comprehend the performance of the modified plates in terms of their sound radiation properties. Detailed discussions on these findings are presented subsequently. It is important to note that the fluid loading parameter $\beta = \frac{\rho _a c_a}{m_s \omega _f}$ plays an important role in determining the effect of a surrounding fluid (such as air or water) on a vibrating structure (e.g., plate)^27,43^. Here, $m_s$ denotes the specific mass of the bare plate, i.e., mass per unit area of the plate. In the present article, since the surrounding fluid is air, and based on the properties of the plate and the frequencies of interest, the fluid loading parameter is less than 1 for the minimum resonant frequencies reported herein (refer to Tables 11 to 14 in the supplementary material). At these frequency ranges the added mass effect due to air on the vibration response of the plate can be safely neglected.

### Validation

In this section, the validation of the semi-analytical approach based on the Rayleigh-Ritz method for vibro-acoustic analysis of wettability-modified plates is presented. A test case using a bare plate (without surface modification), previously examined by Geng et al.^31^, is employed to benchmark the proposed method. This validation focuses on free vibration, forced vibration, and vibro-acoustic features of a rectangular homogeneous isotropic plate with simply supported boundary conditions. It is important to note that, in the current formulation, the bare plate is modeled with denticle mass set to zero. The plate dimensions and material properties are identical to those outlined in Geng et al.^31^ and are listed in Table 3. For the free and forced vibration analysis in this study, $5 \times 5$ mode shapes have been utilized along the $x$ and $y$ directions. It has been ensured that this number of mode shapes results in a converged solution for the response quantities. It’s also important to note that the vibro-acoustic studies are conducted under in-air conditions. The properties of air are defined as follows: air density ($\rho _{a}$) is 1.21 kg/m$^3$, and the sound speed in air ($c_{a}$) is 343 m/s. Furthermore, the loss factor ($\eta$) for the plate is assumed to be 0.001.

Initially, a free vibration analysis is conducted, and the natural frequencies are computed. A comparison with the results from Geng et al.^31^ is performed. The natural frequencies of the first five modes are presented in Table 4. It is observed that the values align closely with Geng et al.^31^, exhibiting a negligible error percentage.

**Table 3:** Geometric and material properties of the plate for validation studies.

Plate properties					
Length (*L*)	Width (*B*)	Thickness (*t*)	Young’s modulus (*E*)	Poisson’s ratio ($\nu$)	Mass density ($\rho$)
m	m	m	GPa		kg/m$^3$
0.40	0.30	0.01	70	0.3	2700

**Table 4:** Validation of structural model: comparison of natural frequencies of the first five modes with Geng et al.^31^.

	Natural frequency (Hz)				
Mode no.	1	2	3	4	5
Present study ($\omega$)	420.20	874.01	1226.98	1630.37	1680.80
Geng et al. ($\omega _r$)	420.20	874.00	1227.00	1630.40	1680.80
Error ($\%$)	0.001	0.001	0.002	0.002	0.000
Error = |$(\omega _r-\omega )/\omega _r\times 100$|					

**Figure 2:**
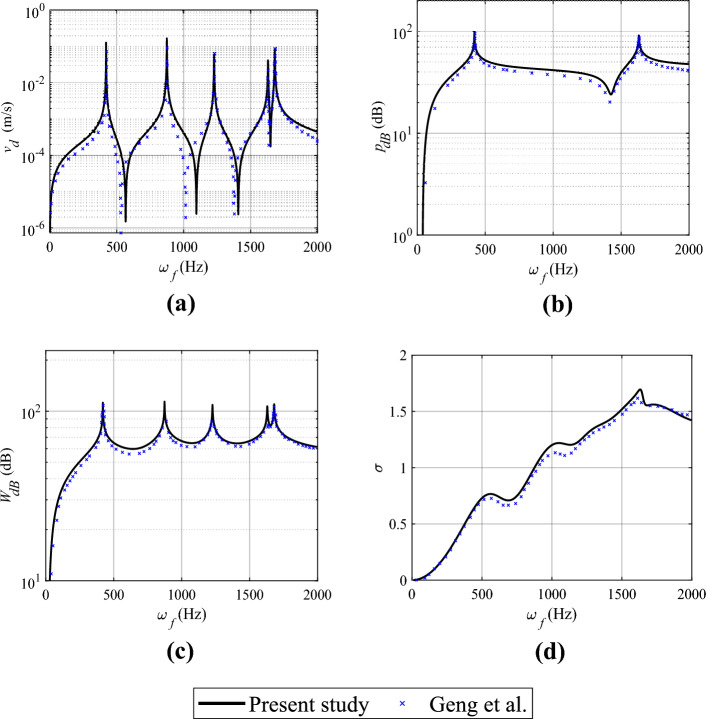
Validation of the vibro-acoustic model. Comparison with Geng et al.^31^: (**a**) driving point velocity (excitation at $x= 0.1$ m and $y= 0.1$ m), (**b**) radiated sound pressure level (at a distance of 3 m ($z= 3$ m) from the centroid of the plate ), (**c**) radiated sound power and , (**d**) radiation efficiency.

Following the free vibration study, a dynamic analysis is conducted by applying a 1 N harmonic point load at (0.10 m, 0.10 m) from the origin on the same plate. Comparisons of the driving point velocity ($v_d$) responses at the harmonic excitation point on the plate are depicted in Fig. 2a. The response curves demonstrate a good agreement, and the overall dynamic characteristics of the results from the proposed method closely align with those from Geng et al.^31^.

**Table 5:** Geometric and material properties of the plate for the test case used in the comparative study.

	Mass ratios $(\sum m_d/m_p)$			Plate properties					
	Spacings								
Material	1	2	5	Length (*L*)	Width (*B*)	Thickness (*h*)	Young’s modulus (*E*)	Poisson’s ratio ($\nu$)	Mass density ($\rho$)
	mm	mm	mm	m	m	m	GPa		$\hbox {kg/m}^3$
Steel	0.079	0.020	0.003	0.500	0.500	0.002	161.9	0.3	7850
Titanium	0.137	0.034	0.006				116	0.32	4506
Aluminium	0.229	0.057	0.009				70	0.35	2700

### Shark skin geometry and material parameters

In this study, wettability-modified plates with shark skin denticle-like surface protrusions over the base plate are examined to explore the impact of these modifications on vibro-acoustic characteristics. The base plate is assumed to be thin, isotropic, and homogeneous. Three different materials for the base plate are considered: steel, aluminium, and titanium. The material and geometric properties of the base plate, including length (*L*), breadth (*B*), thickness (*t*), and material type, are detailed in Table 5.

In the present work, the dimensions of shark skin denticles are chosen based on the works of Domel et al.^44^, wherein denticles were characterized by five parameters (see Fig. 1f, g), viz., (a) chord-wise length ($l_c$), (b) span-wise length ($l_s$), (c) length of side ridges ($l_r$), (d) height of the middle ridge from the plate ($h_1$), and (e) height of the side ridge ($h_2$).

Even though the base plate materials are varied in this parametric study, the density of denticles used in surface modifications is assumed to be made of superaustenitic AL6XN steel and the adopted material properties used in all the calculations are provided in Table 5. Nath et al.^13^ conducted a free vibration analysis of plates using the commercial package ABAQUS^45^, where both the base plate and denticles were considered deformable. They reported that the denticles do not introduce any bending stiffness but contribute solely to the inertia. Therefore, in the proposed semi-analytical formulation, the bending stiffens of the denticle are neglected and they are assumed to be rigid point masses. These denticles/rigid point masses ($m_d$) attached to a bare plate (of mass $m_p=\int _{\Omega }\int _{-h/2}^{h/2} \rho _p \,dz\,d\Omega$) as a two-dimensional array-like pattern ($N \times N$) on both sides of the plate, while maintaining an edge clearance of 5 mm along the boundaries. The spacing between denticles or point masses is varied, and in this study, three different spacings are considered: $L_s = 1$ mm, $L_s = 2$ mm, and $L_s = 5$ mm. The individual mass of a denticle is determined based on the work of Nath et al.^13^, where the denticle design and characterization were conducted using SOLIDWORKS. The mass of the denticles is determined in ABAQUS through a convergence study to estimate a converged value of a single shark skin denticle mass within the finite element method (FEM) framework. This determined mass value is then applied in the current semi-analytical model. Table 5 lists the ratio of the total denticle added mass ($\sum m_d$) to the bare plate mass ($m_p$) for various denticle spacings. Additionally, it is assumed in the present semi-analytical formulation that the denticles (rigid point mass) are firmly attached to the plate in such a way that no relative motion is possible between the plate and the denticle. Among all the spacings, the arrangement with the least spacing shows the most significant changes in vibro-acoustic quantities (Tables 11 to 14 in the supplementary material). For more details on how the plate behaves with the sparser arrangement, please refer to Tables 11 to 14 in the supplementary material.

### Influence on vibro-acoustic parameters

This section investigates the impact of shark skin denticle-like surface modifications on various vibro-acoustic parameters while considering variations in mass ratios. A harmonic analysis is performed to analyze driving point velocity, radiated sound pressure level, radiated sound power, and radiation efficiency. In harmonic analysis, the initial selection of loading points is carefully executed to ensure that these points do not coincide with the node positions of the first six modes for each mass ratio and array spacing. This deliberate positioning is intended to effectively excite all the first six modes. The coordinates of these loading positions ($x_f, y_f$) are tabulated in Table 6. The air medium density ($\rho _{a}$) is assumed to be 1.225 kg/$\hbox {m}^3$, with a sound speed ($c_{a}$) of 340 m/s. Additionally, the loss factor ($\eta$) for the plate is taken as 0.1. For this investigation, six mode shapes along the x direction and six mode shapes along the y direction have been selected. Due to certain boundary conditions, rigid body modes need to be pre-positioned. Accordingly, the first five boundary mode shapes are chosen to ensure convergence in all response measures.

**Table 6:** Excitation points of the applied unit harmonic load.

Excitation point	Boundary conditions							
	SSSS	SCSC	CCFF	CFCF	CFSF	CFFF	CSCG	CCCC
$x_f$	0.10	0.05	0.10	0.10	0.10	0.15	0.10	0.05
$y_f$	0.10	0.25	0.45	0.10	0.10	0.00	0.20	0.15

**Figure 3:**
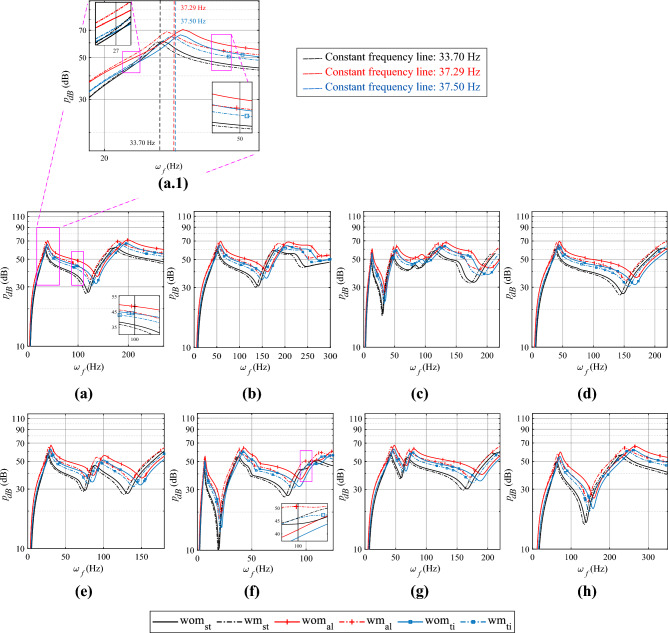
Radiated sound pressure ($p_{dB}$) at 3 m above the centre of gravity of the plate for different boundary conditions : **(a.1)** SSSS: zoomed out to separate two distinct response patterns; **(a)** SSSS; **(b)** SCSC; **(c)** CCFF; **(d)** CFCF; **(e)** CFSF; **(f)** CFFF; **(g)** CSCG; **(h)** CCCC. *wm:* with surface modification, *wom:* without surface modification. Base plate material, *st:* steel, *ti:* titanium, *al:* aluminium.

#### Influence on radiated sound pressure level (SPL)

In this subsection, a comparative study is presented, wherein the sound pressure level (*p*) is compared for plates with and without surface modifications. The comparison involves variations in various boundary conditions and base plate materials. The influence of shark skin wettability modifications on the radiated sound pressure level is assessed by examining and comparing the measurements at a 3-meter normal distance from the central point at the midplane of the plate. Figure 3 depicts the variation of SPL with excitation frequency. In each subfigure, the base plate material is varied while keeping the boundary conditions the same. Subfigures 3a–h present SPL for different boundary conditions.

Firstly, in the comparison of plates without surface modifications for different base plate materials, analysing the values for the first mode under the SSSS boundary condition (see Fig. 3b) reveals that the radiated pressure at the first mode is 23.165 mPa for the heaviest base plate material, steel. Conversely, for the lighter titanium, the SPL is 40.356 mPa ($\sim$ 1.7 times higher), and for the lightest aluminium, it is 67.351 mPa ($\sim$ 2.9 times higher). This increase in SPL for lighter plates is observed for all the boundary conditions considered herein and the ‘bottom-left shift’ trend in SPL can primarily be attributed to the change in mass.

Based on the denticle spacing, the observation reveals that the SPL decreases at resonant frequencies when introducing shark skin modification (maximum at the densest spacing: 1mm) over a plate while keeping the base material constant. For instance, in the case of densest denticle spacing with steel base plate material ($\sum m_d/m_p=$0.079), under SSSS boundary conditions, a reduction in radiated pressure of approximately 7.54$\%$ is observed (the radiated pressure for the base plate is 23.165 mPa, while for the plate with densest spacing surface modification, it measures 21.419 mPa) at resonant frequencies. With an intermediate-mass ratio of $\sum m_d/m_p=0.137$ (titanium base plate), a 12.44$\%$ decrease in SPL values is noted. In contrast, for the lightest aluminium base plate ($\sum m_d/m_p=0.229$), a 19.16$\%$ decrease is reported for the same boundary condition. Among all the studied boundary conditions and base plate materials, the CSCG boundary condition with an aluminium base plate shows the greatest reduction in radiated sound pressure for 1 mm denticle spacing. A reduction of 20.69% is achieved in the fifth mode. Refer to Table 9 of the supplementary material for details.

An overall trend of a bottom-left shift in plots of sound pressure level as a function of loading frequency is observed, occurring in almost all boundary conditions, if shark skin type wettability modifications are introduced (refer to Fig. 3). Moreover, for the densest arrangement, the reduction ranges from 7–8$\%$ for the steel base plate, 11–14$\%$ for the titanium plate, and lastly 18–20$\%$ for the steel base plate with the densest denticle spacing, when measured in Pa scale. This signifies the increasing dependency of the reduction of response amplitude on the mass ratio parameter.

Here, it is crucial to emphasize that although the peak resonant frequencies at all modes are decreasing, at some intermediate excitation frequencies, the radiated pressure level increases due to surface modification. This phenomenon can be attributed to the ‘bottom-left shift’ trend, where the entire plots shift collectively towards low excitation frequency, including a leftward shift in the resonant frequencies. This results in two distinct conditions around the resonant frequency region. In the leftward or lower frequency region (region left to the vertical lines shown in the inset of Fig. 3a.1) of the ‘bottom-left shift’ trend, plates with surface modification exhibit higher values of radiated sound pressure (SPL). On the other hand, on the right or higher frequency side (region right to the vertical lines shown in the inset of the same figure), radiated sound pressure (SPL) become lower under surface treatment. Overlapping vibro-acoustic responses with and without surface modification in the same plot, an isofrequency line can be drawn through the intersection, situated between two consecutive peak resonance amplitudes (one for surface modification and one without), effectively divides the nearby region into two distinct parts. On the right side, corresponding to the higher frequency range, the response of the surface-modified plates becomes lower. Conversely, on the left side, within the lower frequency range, plates with surface modifications exhibit higher frequency responses. It is important to note that there can exist multiple such isofrequency lines for the excitation frequency range considered herein.

In general, the surface modification leads to a bottom-left shift, with higher mass ratios ($\sum m_d/m_p$) resulting in a more pronounced increase in Sound Pressure Level (SPL) on the left side of the resonance frequency and a more significant decrease on the right side (See the zoomed view of in the inset of Fig. 3a.1).

**Figure 4:**
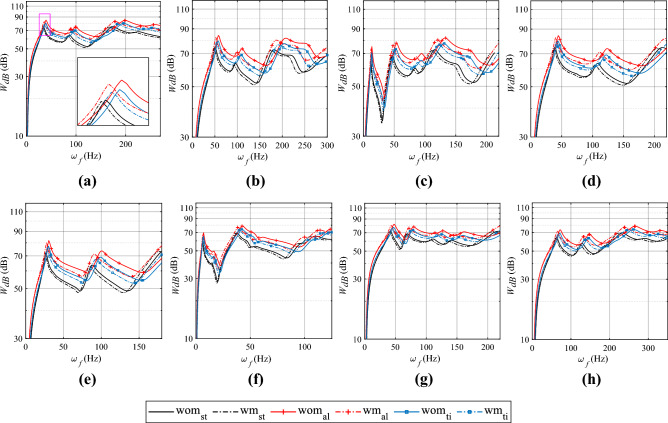
Radiated sound power ($P_{dB}$) of the plate for different boundary conditions : **(a)** SSSS; **(b)** SCSC; **(c)** CCFF; **(d)** CFCF; **(e)** CFSF; **(f)** CFFF; **(g)** CSCG; **(h)** CCCC. *wm:* With surface modification, *wom:* without surface modification. Base plate material; *st:* steel, *ti:* titanium, *al:* aluminium.

#### Influence on sound power level

In Fig. 4, the graph illustrates the variation of radiated power with excitation frequency for plates with and without surface modifications, considering different boundary conditions and base plate materials. Trends in radiated power follow similar trends to other parameters related to vibro-acoustic response. It is noteworthy that introducing additive surface modifications leads to a reduction in radiated sound power at the resonant frequency. For instance, if the base plate material remains the same, and a surface modification is introduced under SSSS boundary conditions for plates with the lowest mass ratio ($\sum m_d/m_p=0.079$), such as steel base plates, an approximate 14.49$\%$ reduction in radiated power is observed (from 0.0364 mW to 0.0312 mW). This reduction increases to 23.29$\%$ (from 0.110 mW to 0.084 mW) for intermediate mass ratios with titanium base plates ($\sum m_d/m_p=0.137$) and further ascends to a more pronounced 34.60$\%$ decrease (from 0.307 mW to 0.201 mW) for the maximum mass ratio with aluminium base plates ($\sum m_d/m_p=0.229$). The decrease in radiated power at resonant frequencies is observed for all other boundary conditions examined in this study. However, it’s worth noting that the percentage reduction in different modes does not exhibit a consistent trend. Furthermore, with the densest arrangement, the reduction varies from 10-22$\%$ for the steel base plate, 20-35$\%$ for the titanium plate, and 30-50$\%$ for the aluminium base plate with the densest denticle spacing when measured in mW scale. Among the various base plate materials and boundary conditions studied, the CSCG boundary condition with an aluminium base plate achieves the highest reduction in radiated power for 1 mm denticle spacing, with a reduction of 47.82% observed in the fifth mode. Refer to Table 9 in the supplementary material for details.

**Figure 5:**
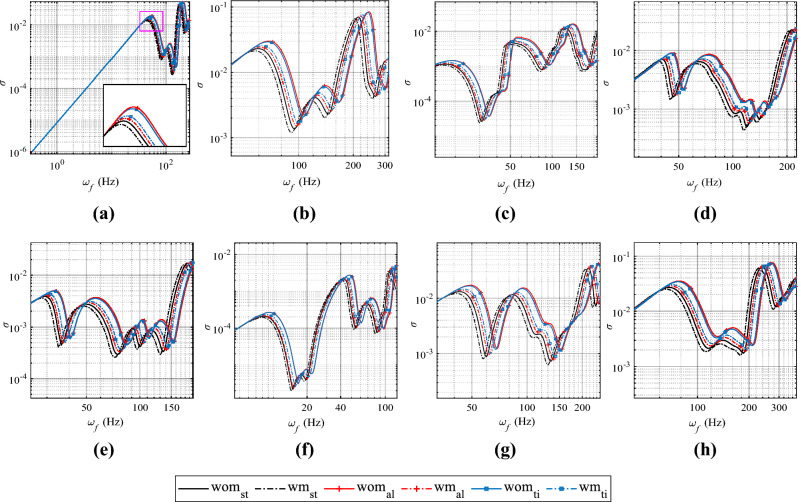
Radiation efficiency ($\sigma$) for different boundary conditions : **(a)** SSSS; **(b)** SCSC; **(c)** CCFF; **(d)** CFCF; **(e)** CFSF; **(f)** CFFF; **(g)** CSCG; **(h)** CCCC. *wm:* With surface modification, *wom:* without surface modification. Base plate material; *st:* steel, *ti:* titanium, *al:* aluminium.

#### Influence on radiation efficiency

In Fig. 5, the graph displays the variation in radiation efficiency within a specified frequency range for different boundary conditions. Each subfigure showcases the radiation efficiency for plates with and without shark skin, while concurrently varying the base plate material, but maintaining the same boundary condition.

The introduction of shark skin denticle-like additive surface modifications seems to lead to a reduction in radiation efficiency, particularly in the low-frequency range. This decrease is emphasized in Fig. 5a for the SSSS boundary condition (as shown in the inset of the figure). Additionally, it is evident that the reduction in radiation efficiency is most pronounced for the largest mass ratio ($\sum m_d/m_p=0.229$), as indicated by the maximum leftward shift due to shark skin denticle-like modifications. Nevertheless, at certain high frequencies (above 100 Hz), there appears to be an increase in radiation efficiency. However, at higher frequencies, this behaviour flips, and a further shift to the bottom left is observed. This pattern reversal at higher frequencies contributes to the observed fluctuations in radiation efficiency. Similar bottom-left shift patterns are noticed (Fig. 5a–h) for all other boundary conditions. However, the specific position of the shift varies, leading to distinct frequencies at which the pattern reversal or fluctuations in radiation efficiency occur.

Specifically, focusing on plates with SSSS boundary conditions in the first mode, a significant 19.10$\%$ reduction in radiation efficiency is observed for plates with surface modification compared to bare aluminium base plates, particularly for the largest mass ratio, $\sum m_d/m_p=0.229$. As the mass ratios decrease, the reduction in radiation efficiency is approximately 12.39$\%$ when additive surface modification with shark skin is applied to titanium base plates ($\sum m_d/m_p=0.137$), and 7.52$\%$ for surface modifications on steel plates ($\sum m_d/m_p=0.079$).

In comparison to cases with plates lacking surface modifications, the radiation efficiency for the steel bare plate is approximately 0.01046. For the titanium plate, it is 0.01329, indicating a 27.05$\%$ increase in radiation efficiency, while for aluminium, it is 0.01369, reflecting a 30.87$\%$ increase in radiation efficiency. Additionally, the percentage reduction in radiation efficiency across mode shapes for a given boundary condition appears to exhibit variability and fluctuation when surface modifications are introduced. At a maximum reduction of 35.09%, the radiation efficiency is observed under the CSCG boundary condition with an aluminium base plate for 1 mm denticle spacing. This reduction occurs in the fifth mode. Refer to Table 9 in the supplementary material for details.

#### Influence on the directivity of radiated sound pressure

**Figure 6:**
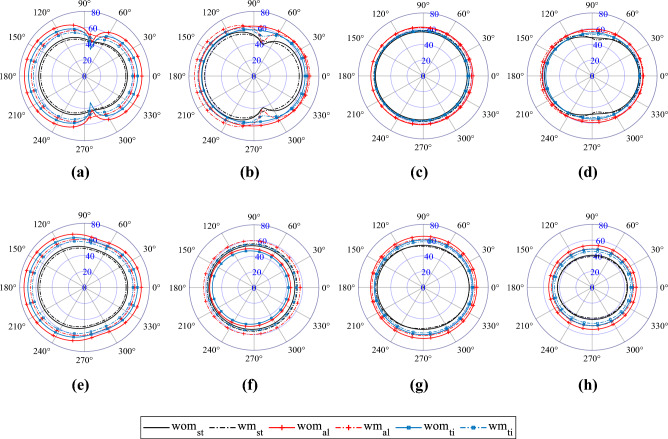
Directivity of radiated sound pressure for an excitation frequency of 100 HZ different boundary conditions: **(a)** SSSS; **(b)** SCSC; **(c)** CCFF; **(d)** CFCF; **(e)** CFSF; **(f)** CFFF; **(g)** CSCG; **(h)** CCCC. SPL levels are marked in dB along the radial axis. *wm:* With surface modification, *wom:* without surface modification. Base plate material; *st:* steel, *ti:* titanium, *al:* aluminium.

Directivity of radiated sound pressure from baffled rectangular plates for different boundary conditions is presented in Figs. 6a-h . Similar to the previously discussed vibro-acoustic quantities in the preceding section, a subfigure illustrates the directivity of radiated sound pressure for plates with identical boundary conditions but varying base plate materials, including cases with and without surface modification. In each of the subfigures, the boundary condition is varied to investigate its influence on the directivity of radiated sound pressure. In these plots, the maximum steady-state radiated sound pressure response at a radial distance of 1 m from the centre of mass of the bare plate is presented on the $xy$-plane for angles ranging from 0$^\circ$ to 360$^\circ$. The plates are excited with an operating frequency of 100 Hz and a 1 N load applied at various excitation points, as specified in Table 6.

In Fig. 6a, the radiated pressure level is depicted for plates featuring the SSSS boundary condition. Notably, the application of shark skin-type surface modifications on steel base plates ($\sum m_d/m_p=0.079$) results in a distinct reduction in SPL. Furthermore, the introduction of a lighter base plate material, such as titanium ($\sum m_d/m_p=0.137$), with the same arrangement of denticles, leads to a higher percentage decrease in SPL compared to the base plate alone. Moreover, utilizing an even lighter base plate material like aluminium ($\sum m_d/m_p=0.229$) results in a more pronounced percentage reduction in SPL, especially at the low excitation frequency of 100 Hz. When comparing the base plates without any surface modifications, the radiated sound power level is higher for the lighter plates. This pattern is well-justified by the earlier explanations: as the plate becomes lighter, it displays greater movement, resulting in higher radiated pressure levels. However, with the same surface denticle positioning, $\sum m_d$ remains constant, while the denominator varies in the mass ratio term. For the lighter base plate material, this results in a higher mass ratio ($\sum m_d/m_p$). A higher mass ratio corresponds to a more pronounced reduction in this case. However, in SCSC boundary conditions (see Fig. 6b), an amplification in the radiated sound pressure is noted for the aluminium base plate upon utilization of surface modifications. This observation is aligned with the discussion presented for the isofrequency lines in previous section. The amplification or reduction of the vibro-acoustic response quantities depends on the presence of the nearby isofrequency lines lying within the nearby free vibration modes of bare and surface-modified plates. As the mass ratio increases, the reduction or amplification of the radiated sound pressure becomes more prominent and depends on the presence of nearby iso-frequencies. This observation holds for Fig. 6b–h as well.

However, it is important to note that when excited at a certain frequency, a unique caving-in pattern appears at different angles due to the presence of nearby free vibration modes. For example, this distinctive pattern becomes prominent in Fig. 6a, nearly at $\pm 80^\circ$. Three distinct observations can be made here: Firstly, for the steel base plate, no caving-in patterns are observed for both bare and surface-modified plates. In this case, the nearby modes for bare and surface-modified plates are 86.136 Hz and 82.826 Hz, respectively. These frequencies are far from the excitation frequency, causing less excitation of the nearby modes by the applied harmonic loading for steel plates. Secondly, for the lightest base plate, aluminium, a significant caving-in pattern is observed in the bare plate, whereas for the surface-modified plates, this pattern becomes insignificant. This is because, if the nearby free vibration modes are considered, distinct modes are present at 98.590 Hz (2nd mode) for the bare aluminium plate. Subsequently, after a 1 mm spacing surface treatment, the nearby free vibration mode shifts to 88.645 Hz (2nd mode), pushing it much away from the excitation frequency of 100 Hz. As a result, the loading excites the second mode of the bare plate largely compared to the second mode of the surface-modified plate. Lastly, in Fig. 6b, although the aluminium base plate does not show any caving-in pattern, a distinct caving-in can be observed in the plate with surface modifications. This is because, for the aluminium base plate with SCSC boundary conditions, the bare plate has a nearby free vibration mode at 109.470 Hz (2nd mode). In contrast, the surface-modified plate with 1 mm spacing has a nearby mode at 98.427 Hz (2nd mode), which is much closer to the excitation frequency of 100 Hz. Overall, the proximity of the excitation frequency to the nearby modes results in a distinct caving-in pattern in the directivity plots. These observations hold for all the directivity plots presented in Fig. 6a–h for various boundary conditions, base plate materials, and surface modifications. The interplay of multimode excitation and dominant resonant frequencies can significantly impact the sound pressure level (SPL) and subsequently the directivity patterns.

This suggests that a distributed array of denticles used for surface modification does not universally weaken the acoustic radiation characteristics of the structure. While the structure may behave like a weaker radiator with surface modifications at resonance frequencies, this effect does not hold across a broader frequency spectrum. A more accurate representation of the model can be achieved by considering the elastic nature of the denticles, a consideration that could demand high-fidelity numerical calculations^13^.

## Conclusions

This study extensively explores the vibro-acoustic characteristics of a thin, isotropic, and homogeneous plate featuring bio-inspired shark skin type additive surface modifications. A semi-analytical approach, utilizing the Rayleigh-Ritz formulation, is developed to analyze the vibrations of these plates. The sound radiation characteristics are then derived from the solution of the Rayleigh integral. In this investigation, the shark skin dermal denticles are represented as point masses on both the upper and lower surfaces of a plate, organized in a bi-directional arrayed pattern. The research delves into the examination of both free and forced vibration responses of these plates, taking into account various array spacings, boundary conditions, and base plate materials. The study examines the vibro-acoustic properties of plates with bio-inspired shark skin surfaces, emphasizing the impact of additive surface modifications on radiated sound pressure, sound power, and radiation efficiency. A few noteworthy observations on the vibro-acoustic response of plates with shark skin type additive surface modifications with denticles are summarized as follows:

- The incorporation of a patterned bi-directional array of denticle masses results in a decrease in the natural frequency with minimal changes in mode shapes. Moreover, it reduces the response/sound-radiation characteristics of the plate at the resonance frequencies.
- The most substantial reduction in natural frequency and peak responses at resonance occurs with the densest denticle considered herein (1 mm spacing in both directions), displaying the maximum mass ratio, i.e., the ratio of denticle mass to the bare plate mass. Higher mass ratios correspond to more significant reductions in vibro-acoustic quantities at resonance. Additionally, increasing mass ratios result in a broader shift or more pronounced reduction in radiation efficiencies in the frequency spectrum.
- An overall trend of ‘bottom-left shift’, i.e., shift towards a lower frequency as well as a reduction in peak value is observed in frequency responses (in the cases of radiated pressure and radiated power) with the increase in denticle spacing.
- The lightest material base plate material considered in this study (viz, aluminium, 1mm denticle spacing), exhibits the most significant reduction in the amplitude of frequency response quantities at resonant frequencies. Conversely, the reduction becomes less pronounced as the mass ratio decreases.
- The inclusion of surface modifications on both sides results in a notable reduction in vibro-acoustic parameters in comparison to a bare plate; a maximum reduction of 20.69$\%$ in radiated pressure, 47.82$\%$ in radiated power, and 35.09$\%$ in radiation efficiency are observed for the densest denticle spacing across various boundary conditions and base plate materials.
- Comparing frequency response and vibro-acoustic responses between plates with surface modification to those without, an isofrequency line emerges, delineating the nearby region into two distinct parts. In the higher frequency range on the right side of this line, the response of surface-modified plates decreases, while on the left side in the lower frequency range, plates with surface modifications demonstrate higher frequency responses. This suggests that the structure with surface modifications may behave as a weaker radiator specifically at resonance frequencies.

Future research extensions could explore the vibro-acoustic characteristics of sharkskin-inspired denticle surface modifications underwater. Although it is known that free vibration frequencies decrease due to the hydrodynamic added mass, the corresponding changes in vibro-acoustic properties need to be explored. Future work could first explore the effects of denticle stiffness to better understand the dynamic behaviour of these surface modifications. Once this is established, integrating electrorheological fluid into phononic crystals (PCs) could be examined, focusing on elastic deformation using electro/magnetic pulses during vibration. This phased approach could lead to the development of an acoustic meta-model with enhanced power modulation potential. These findings could significantly improve the design of marine installations with vibrating machinery components and have applications in noise engineering, aerospace, and the automobile industry (Supplementary Information).

## Supplementary Information

Supplementary Information.

## Data Availability

The data that support the findings is provided within the manuscript or supplementary information files.
