# Supplementary material for: Acoustic radiation characteristics of shark skin inspired surface modified plates
Source: Sci Rep. 2024 Oct 9;14:23639. doi: 10.1038/s41598-024-72489-7 (PMC11464837; doi:10.1038/s41598-024-72489-7)
Supplement: Supplementary file 1 — Supplementary Information. [file 41598_2024_72489_MOESM1_ESM.pdf]

Acoustic Radiation Characteristics of Shark Skin Inspired Surface Modified Plates

Aninda Pal[1]
[1]Dept of Ocean Engineering & Naval Architecture
IIT Kharagpur, Kharagpur, 721302, West Bengal, India
aninda.buzz@gmail.com

Ritwik Ghoshal[1*]
[1*]Dept of Ocean Engineering & Naval Architecture
IIT Kharagpur, Kharagpur, 721302, West Bengal, India
ritwik@naval.iitkgp.ac.in

July 25, 2024

Non-dimensional Frequency Parameter

The natural frequencies are reported using a non-dimensional frequency parameter (Ω),

$$\bar{\Omega} = \sqrt{\omega^2 \rho_p h L^2 B^2 / D}. \quad (1)$$

Where,

$$\begin{aligned}
\omega &= \text{dimensional natural frequency}(rad/s),\\
\rho_p &= \text{density of the plate } (kg/m^3),\\
h &= \text{height of the plate } (m),\\
L &= \text{length of the plate } (m),\\
B &= \text{width of the plate } (m),\\
D &= \text{bending stiffness of the plate} = \frac{Eh^3}{12(1-\nu^2)}(N.m),\\
E &= \text{Young's modulus of the plate material } (N/m^2) \text{ and},\\
\nu &= \text{Poisson's ratio .}
\end{aligned}$$

Supplementary Tables

Table 1: Non-dimensional natural frequency ($\bar{\Omega}$) for different base plate materials and different boundary conditions, SSSS, SCSC, CCFF, and CFCF.

	$\bar{\Omega}_{\text{wom}}$	Steel $\bar{\Omega}^{\text{st}}_{\text{wm}}$	% Change	Titanium $\bar{\Omega}^{\text{ti}}_{\text{wm}}$	% Change	Aluminium $\bar{\Omega}^{\text{al}}_{\text{wm}}$	% Change	Mode sequence
SSSS	19.74	18.98	3.84%	18.47	6.42%	17.75	10.09%	1
	49.35	47.45	3.84%	46.18	6.42%	44.37	10.09%	2
	49.35	47.45	3.84%	46.18	6.42%	44.37	10.09%	3
	78.96	75.92	3.84%	73.89	6.42%	70.99	10.09%	4
	98.70	94.90	3.84%	92.36	6.42%	88.74	10.09%	5
	98.70	94.90	3.84%	92.36	6.42%	88.74	10.09%	6
	128.31	123.38	3.84%	120.07	6.42%	115.37	10.08%	7
	128.31	123.38	3.84%	120.07	6.42%	115.37	10.08%	8
	167.78	161.34	3.84%	157.01	6.42%	150.87	10.08%	9
	167.78	161.34	3.84%	157.01	6.42%	150.87	10.08%	10
SCSC	28.96	27.84	3.84%	27.10	6.42%	26.04	10.09%	1
	54.79	52.69	3.84%	51.27	6.42%	49.27	10.09%	2
	69.33	66.67	3.84%	64.88	6.42%	62.34	10.09%	3
	94.65	91.01	3.84%	88.57	6.42%	85.10	10.09%	4
	102.35	98.42	3.84%	95.78	6.42%	92.03	10.09%	5
	129.11	124.15	3.84%	120.82	6.42%	116.09	10.09%	6
	140.42	135.03	3.84%	131.40	6.42%	126.26	10.08%	7
	154.97	149.02	3.84%	145.02	6.42%	139.34	10.08%	8
	170.58	164.03	3.84%	159.63	6.42%	153.38	10.08%	9
	200.46	192.76	3.84%	187.59	6.42%	180.24	10.08%	10
CCFF	6.95	6.70	3.62%	6.49	6.06%	6.19	9.55%	1
	24.07	23.20	3.58%	22.47	6.00%	21.43	9.46%	2
	26.73	25.75	3.66%	25.08	6.12%	24.13	9.64%	3
	47.99	46.27	3.60%	44.86	6.03%	42.84	9.50%	4
	63.12	60.84	3.62%	59.12	6.07%	56.68	9.55%	5
	65.95	63.56	3.63%	61.96	6.09%	59.69	9.59%	6
	86.15	83.06	3.58%	80.65	6.01%	77.20	9.47%	7
	89.19	85.95	3.63%	83.52	6.08%	80.05	9.58%	8
	122.12	117.71	3.62%	114.56	6.06%	110.06	9.54%	9
	124.74	120.21	3.63%	117.14	6.07%	112.73	9.55%	10
CFCF	22.30	21.46	3.77%	20.89	6.30%	20.07	9.90%	1
	26.54	25.57	3.66%	24.82	6.12%	23.75	9.64%	2
	43.82	42.24	3.60%	40.99	6.04%	39.21	9.52%	3
	61.54	59.23	3.76%	57.65	6.29%	55.41	9.89%	4
	67.57	65.09	3.66%	63.29	6.13%	60.73	9.65%	5
	80.11	77.24	3.59%	75.12	6.02%	72.10	9.49%	6
	88.39	85.20	3.61%	82.70	6.05%	79.14	9.53%	7
	120.75	116.21	3.76%	113.13	6.29%	108.76	9.88%	8
	125.50	121.01	3.58%	117.53	6.01%	112.57	9.48%	9
	127.50	122.83	3.66%	119.53	6.13%	114.84	9.65%	10

$\bar{\Omega}^{\text{i}}_{\text{wm}}$: Nondimensional frequencies with surface modification; i $\in$ (st,ti,al);
st: Steel ($\sum m_d/m_p = 0.079$), ti:Titanium ($\sum m_d/m_p = 0.137$), al: Aluminium ($\sum m_d/m_p = 0.229$)
$\bar{\Omega}_{\text{wom}}$: Nondimensional frequencies without surface modification; % change = $(\bar{\Omega}_{\text{wom}} - \bar{\Omega}^{\text{i}}_{\text{wm}})/\bar{\Omega}_{\text{wom}}$

Table 2: Non-dimensional natural frequency parameter ($\bar{\Omega}$) for different base materials and different boundary conditions, CFSF, CFFF, CSCG, and CCCC.

	$\bar{\Omega}_{\rm wom}$	Steel $\bar{\Omega}^{\rm st}_{\rm wm}$	% Change	Titanium $\bar{\Omega}^{\rm ti}_{\rm wm}$	% Change	Aluminium $\bar{\Omega}^{\rm al}_{\rm wm}$	% Change	Mode sequence
CFSF	15.32	14.74	3.77%	14.34	6.30%	13.77	9.90%	1
	20.68	19.92	3.66%	19.30	6.12%	18.41	9.64%	2
	39.90	38.46	3.60%	37.30	6.04%	35.65	9.52%	3
	49.81	47.94	3.76%	46.66	6.29%	44.83	9.89%	4
	56.64	54.56	3.66%	53.02	6.13%	50.81	9.65%	5
	77.49	74.71	3.59%	72.66	6.02%	69.74	9.49%	6
	79.29	76.43	3.61%	74.13	6.05%	70.86	9.53%	7
	104.08	100.17	3.76%	97.51	6.29%	93.73	9.88%	8
	111.46	107.38	3.66%	104.47	6.13%	100.32	9.65%	9
	118.19	113.95	3.58%	110.65	6.01%	105.94	9.48%	10
CFFF	3.50	3.37	3.65%	3.28	6.12%	3.16	9.63%	1
	8.53	8.23	3.56%	7.94	5.97%	7.53	9.41%	2
	21.49	20.71	3.62%	20.11	6.07%	19.25	9.55%	3
	27.43	26.46	3.54%	25.75	5.93%	24.74	9.36%	4
	31.20	30.09	3.54%	29.15	5.94%	27.79	9.36%	5
	55.22	53.29	3.49%	51.59	5.86%	49.15	9.25%	6
	61.86	59.60	3.65%	58.08	6.11%	55.91	9.62%	7
	64.63	62.36	3.51%	60.62	5.89%	58.10	9.29%	8
	71.58	69.05	3.54%	67.23	5.94%	64.64	9.36%	9
	94.22	90.92	3.50%	88.28	5.87%	84.49	9.26%	10
CSCG	23.82	22.92	3.78%	22.31	6.32%	21.45	9.93%	1
	39.11	37.63	3.78%	36.64	6.32%	35.23	9.93%	2
	63.53	61.14	3.78%	59.52	6.32%	57.22	9.93%	3
	75.93	73.06	3.78%	71.13	6.32%	68.39	9.93%	4
	79.55	76.55	3.78%	74.52	6.32%	71.65	9.93%	5
	114.91	110.57	3.78%	107.65	6.32%	103.50	9.93%	6
	122.93	118.29	3.78%	115.16	6.32%	110.72	9.93%	7
	133.92	128.87	3.78%	125.47	6.32%	120.63	9.93%	8
	139.70	134.42	3.78%	130.87	6.32%	125.82	9.93%	9
	171.17	164.71	3.77%	160.36	6.32%	154.18	9.93%	10
CCCC	36.01	34.62	3.84%	33.69	6.42%	32.37	10.09%	1
	73.46	70.64	3.84%	68.74	6.42%	66.05	10.09%	2
	73.46	70.64	3.84%	68.74	6.42%	66.05	10.09%	3
	108.38	104.21	3.84%	101.42	6.42%	97.45	10.09%	4
	131.77	126.71	3.84%	123.31	6.42%	118.48	10.09%	5
	132.33	127.25	3.84%	123.83	6.42%	118.98	10.09%	6
	165.46	159.11	3.84%	154.84	6.42%	148.78	10.09%	7
	165.46	159.11	3.84%	154.84	6.42%	148.78	10.09%	8
	210.76	202.67	3.84%	197.23	6.42%	189.51	10.08%	9
	210.76	202.67	3.84%	197.23	6.42%	189.51	10.08%	10

$\bar{\Omega}^{\rm i}_{\rm wm}$: Nondimensional frequencies with surface modification; i $\in$ (st,al,ti);
st: Steel ($\sum m_d/m_p = 0.079$), al: Aluminium ($\sum m_d/m_p = 0.229$), ti:Titanium ($\sum m_d/m_p = 0.137$)
$\bar{\Omega}_{\rm wom}$: Nondimensional frequencies without surface modification; % change = $(\bar{\Omega}_{\rm wom} - \bar{\Omega}^{\rm i}_{\rm wm})/\bar{\Omega}_{\rm wom}$

Table 3: vibro-acoustic quantities at resonant frequencies for different base materials; boundary condition: SSSS.

		Mode no.	Unit	1	2	3	4	5
Steel base ($\sum m_d/m_p$ =0.079)	wom	Frequency (ω)	Hz	34.454	86.139	86.142	137.827	172.284
		Velocity (v_d)	mm/s	5.884	12.226	12.225	10.409	6.904
		Pressure (p)	mPa	23.165	1.889	1.889	2.132	25.005
		Power (W)	mW	0.03641	0.00273	0.00273	0.00051	0.03401
		ERP (W_{ERP})	mW	3.48200	2.93900	2.93900	1.56700	0.85500
		Radiation efficiency (σ)		0.01046	0.00093	0.00093	0.00032	0.03979
	wm	Frequency (ω)	Hz	33.130	82.829	82.833	132.533	165.667
		Velocity (v_d)	mm/s	5.658	11.757	11.756	10.009	6.639
		Pressure (p)	mPa	21.419	1.747	1.746	1.972	23.123
		Power (W)	mW	0.03114	0.00217	0.00217	0.00041	0.02960
		ERP (W_{ERP})	mW	3.21900	2.71800	2.71800	1.44900	0.79000
		Radiation efficiency (σ)		0.00967	0.00080	0.00080	0.00028	0.03746
	% reduction in velocity			3.84%	3.84%	3.84%	3.84%	3.84%
	% reduction in radiated pressure			7.54%	7.53%	7.53%	7.49%	7.53%
	% reduction in radiated power			14.49%	20.27%	20.27%	18.77%	12.96%
	% reduction in ERP			7.54%	7.54%	7.54%	7.54%	7.54%
	% reduction in Radiation efficiency			7.52%	13.78%	13.78%	12.16%	5.87%
Titanium base ($\sum m_d/m_p$ =0.137)	wom	Frequency (ω)	Hz	38.854	97.140	97.143	155.428	194.286
		Velocity (v_d)	mm/s	9.090	18.888	18.886	16.079	10.665
		Pressure (p)	mPa	40.356	3.290	3.290	3.708	43.548
		Power (W)	mW	0.11041	0.01027	0.01028	0.00186	0.09674
		ERP (W_{ERP})	mW	8.30900	7.01400	7.01500	3.74000	2.04000
		Radiation efficiency (σ)		0.01329	0.00146	0.00146	0.00050	0.04743
	wm	Frequency (ω)	Hz	36.358	90.900	90.904	145.447	181.810
		Velocity (v_d)	mm/s	8.506	17.674	17.673	15.047	9.980
		Pressure (p)	mPa	35.338	2.881	2.881	3.250	38.141
		Power (W)	mW	0.08470	0.00699	0.00699	0.00127	0.07704
		ERP (W_{ERP})	mW	7.27600	6.14200	6.14300	3.27500	1.78600
		Radiation efficiency (σ)		0.01164	0.00114	0.00114	0.00039	0.04313
	% reduction in velocity			6.42%	6.42%	6.42%	6.42%	6.42%
	% reduction in radiated pressure			12.44%	12.43%	12.43%	12.35%	12.42%
	% reduction in radiated power			23.29%	31.99%	31.99%	31.36%	20.37%
	% reduction in ERP			12.44%	12.43%	12.43%	12.43%	12.43%
	% reduction in Radiation efficiency			12.39%	22.33%	22.33%	21.62%	9.07%
Aluminium base ($\sum m_d/m_p$ =0.229)	wom	Frequency (ω)	Hz	39.436	98.593	98.597	157.755	197.193
		Velocity (v_d)	mm/s	14.947	31.057	31.055	26.439	17.536
		Pressure (p)	mPa	67.351	5.491	5.490	6.187	72.674
		Power (W)	mW	0.30747	0.02940	0.02940	0.00531	0.26699
		ERP (W_{ERP})	mW	22.46500	18.96400	18.96500	10.11100	5.51400
		Radiation efficiency (σ)		0.01369	0.00155	0.00155	0.00053	0.04842
	wm	Frequency (ω)	Hz	35.457	88.648	88.651	141.844	177.306
		Velocity (v_d)	mm/s	13.439	27.923	27.921	23.772	15.767
		Pressure (p)	mPa	54.446	4.439	4.439	5.009	58.771
		Power (W)	mW	0.20110	0.01585	0.01586	0.00291	0.18527
		ERP (W_{ERP})	mW	18.16000	15.33100	15.33200	8.17400	4.45800
		Radiation efficiency (σ)		0.01107	0.00103	0.00103	0.00036	0.04156
	% reduction in velocity			10.09%	10.09%	10.09%	10.09%	10.09%
	% reduction in radiated pressure			19.16%	19.15%	19.15%	19.04%	19.13%
	% reduction in radiated power			34.60%	46.06%	46.06%	45.17%	30.61%
	% reduction in ERP			19.16%	19.16%	19.16%	19.16%	19.15%
	% reduction in Radiation efficiency			19.10%	33.28%	33.28%	32.18%	14.17%

wm: With surface modification; wom: Without surface modification;

Table 4: vibro-acoustic quantities at resonant frequencies for different base materials; boundary condition: SCSC.

		Mode no.	Unit	1	2	3	4	5
Steel base ($\sum m_d/m_p$ =0.079)	wom	Frequency (ω)	Hz	50.543	95.645	121.025	165.219	178.661
		Velocity (v_d)	mm/s	4.010	7.078	0.486	3.939	6.977
		Pressure (p)	mPa	21.356	2.479	1.531	6.085	18.114
		Power (W)	mW	0.03085	0.00216	0.00035	0.00212	0.01793
		ERP (W_{ERP})	mW	1.60300	1.55300	0.08500	0.24100	0.82100
		Radiation efficiency (σ)		0.01925	0.00139	0.00410	0.00880	0.02185
	wm	Frequency (ω)	Hz	48.601	91.970	116.375	158.872	171.799
		Velocity (v_d)	mm/s	3.856	6.806	0.467	3.788	6.709
		Pressure (p)	mPa	19.746	2.292	1.416	5.628	16.752
		Power (W)	mW	0.02638	0.00173	0.00029	0.00184	0.01560
		ERP (W_{ERP})	mW	1.48200	1.43600	0.07800	0.22300	0.75900
		Radiation efficiency (σ)		0.01781	0.00121	0.00364	0.00825	0.02055
	% reduction in velocity			3.84%	3.84%	3.84%	3.84%	3.84%
	% reduction in radiated pressure			7.54%	7.54%	7.52%	7.51%	7.52%
	% reduction in radiated power			14.47%	19.60%	17.87%	13.34%	13.02%
	% reduction in ERP			7.54%	7.54%	7.54%	7.54%	7.53%
	% reduction in Radiation efficiency			7.50%	13.04%	11.18%	6.28%	5.93%
Titanium base ($\sum m_d/m_p$ =0.137)	wom	Frequency (ω)	Hz	56.998	107.860	136.480	186.318	201.477
		Velocity (v_d)	mm/s	6.194	10.935	0.751	6.084	10.778
		Pressure (p)	mPa	37.204	4.319	2.665	10.590	31.543
		Power (W)	mW	0.09347	0.00794	0.00120	0.00615	0.05116
		ERP (W_{ERP})	mW	3.82400	3.70500	0.20200	0.57600	1.95900
		Radiation efficiency (σ)		0.02444	0.00214	0.00595	0.01067	0.02612
	wm	Frequency (ω)	Hz	53.336	100.931	127.714	174.352	188.539
		Velocity (v_d)	mm/s	5.796	10.232	0.703	5.694	10.086
		Pressure (p)	mPa	32.578	3.782	2.335	9.277	27.629
		Power (W)	mW	0.07174	0.00547	0.00086	0.00485	0.04066
		ERP (W_{ERP})	mW	3.34900	3.24500	0.17700	0.50500	1.71500
		Radiation efficiency (σ)		0.02142	0.00168	0.00484	0.00960	0.02371
	% reduction in velocity			6.42%	6.42%	6.43%	6.42%	6.42%
	% reduction in radiated pressure			12.44%	12.43%	12.41%	12.39%	12.41%
	% reduction in radiated power			23.25%	31.14%	28.80%	21.20%	20.52%
	% reduction in ERP			12.44%	12.43%	12.44%	12.44%	12.43%
	% reduction in Radiation efficiency			12.35%	21.37%	18.68%	10.01%	9.24%
Aluminium base ($\sum m_d/m_p$ =0.229)	wom	Frequency (ω)	Hz	57.851	109.474	138.523	189.106	204.492
		Velocity (v_d)	mm/s	10.185	17.980	1.235	10.004	17.722
		Pressure (p)	mPa	62.090	7.208	4.448	17.670	52.639
		Power (W)	mW	0.26027	0.02266	0.00341	0.01701	0.14126
		ERP (W_{ERP})	mW	10.34000	10.01800	0.54700	1.55800	5.29500
		Radiation efficiency (σ)		0.02517	0.00226	0.00623	0.01092	0.02668
	wm	Frequency (ω)	Hz	52.014	98.430	124.549	170.033	183.869
		Velocity (v_d)	mm/s	9.158	16.166	1.110	8.995	15.935
		Pressure (p)	mPa	50.193	5.827	3.598	14.296	42.574
		Power (W)	mW	0.17035	0.01247	0.00198	0.01161	0.09773
		ERP (W_{ERP})	mW	8.35900	8.09900	0.44200	1.25900	4.28100
		Radiation efficiency (σ)		0.02038	0.00154	0.00448	0.00922	0.02283
	% reduction in velocity			10.09%	10.09%	10.09%	10.09%	10.09%
	% reduction in radiated pressure			19.16%	19.16%	19.12%	19.09%	19.12%
	% reduction in radiated power			34.55%	44.98%	41.93%	31.75%	30.81%
	% reduction in ERP			19.16%	19.16%	19.16%	19.16%	19.15%
	% reduction in Radiation efficiency			19.04%	31.94%	28.16%	15.57%	14.42%

wm: With surface modification; wom: Without surface modification;

Table 5: vibro-acoustic quantities at resonant frequencies for different base materials; boundary condition: CCFF.

		Mode no.	Unit	1	2	3	4	5
Steel base ($\sum m_d/m_p$ =0.079)	wom	Frequency (ω)	Hz	12.126	42.008	46.667	83.784	110.196
		Velocity (v_d)	mm/s	2.746	8.133	3.929	5.873	10.526
		Pressure (p)	mPa	7.487	3.186	9.945	3.570	11.271
		Power (W)	mW	0.00382	0.00083	0.00675	0.00103	0.00898
		ERP (W_{ERP})	mW	4.58400	3.88100	1.73300	1.29600	1.82800
		Radiation efficiency (σ)		0.00083	0.00021	0.00389	0.00079	0.00491
	wm	Frequency (ω)	Hz	11.687	40.504	44.961	80.768	106.203
		Velocity (v_d)	mm/s	2.648	7.847	3.795	5.673	10.110
		Pressure (p)	mPa	6.958	2.957	9.161	3.264	10.523
		Power (W)	mW	0.00330	0.00070	0.00573	0.00085	0.00779
		ERP (W_{ERP})	mW	4.26100	3.61100	1.61300	1.20700	1.69100
		Radiation efficiency (σ)		0.00077	0.00019	0.00355	0.00070	0.00460
	% reduction in velocity			3.57%	3.51%	3.39%	3.41%	3.95%
	% reduction in radiated pressure			7.07%	7.18%	7.88%	8.58%	6.63%
	% reduction in radiated power			13.63%	14.78%	15.16%	17.09%	13.31%
	% reduction in ERP			7.05%	6.95%	6.97%	6.80%	7.50%
	% reduction in Radiation efficiency			7.08%	8.41%	8.80%	11.05%	6.28%
Titanium base ($\sum m_d/m_p$ =0.137)	wom	Frequency (ω)	Hz	13.596	47.065	52.605	93.971	123.913
		Velocity (v_d)	mm/s	4.228	12.529	5.906	9.049	16.123
		Pressure (p)	mPa	12.971	5.276	17.526	6.320	18.874
		Power (W)	mW	0.01147	0.00241	0.02097	0.00334	0.02576
		ERP (W_{ERP})	mW	10.96200	9.30000	4.05200	3.10300	4.32400
		Radiation efficiency (σ)		0.00105	0.00026	0.00518	0.00108	0.00596
	wm	Frequency (ω)	Hz	12.772	44.241	49.383	88.305	116.394
		Velocity (v_d)	mm/s	3.975	11.790	5.563	8.533	15.058
		Pressure (p)	mPa	11.454	4.649	15.253	5.430	16.796
		Power (W)	mW	0.00895	0.00182	0.01588	0.00243	0.02014
		ERP (W_{ERP})	mW	9.68200	8.22800	3.58000	2.75400	3.78800
		Radiation efficiency (σ)		0.00092	0.00022	0.00444	0.00088	0.00532
	% reduction in velocity			5.98%	5.90%	5.81%	5.70%	6.61%
	% reduction in radiated pressure			11.69%	11.90%	12.97%	14.09%	11.01%
	% reduction in radiated power			22.02%	24.19%	24.28%	27.34%	21.84%
	% reduction in ERP			11.67%	11.52%	11.64%	11.25%	12.40%
	% reduction in Radiation efficiency			11.71%	14.32%	14.30%	18.14%	10.77%
Aluminium base ($\sum m_d/m_p$ =0.229)	wom	Frequency (ω)	Hz	13.678	47.289	53.350	94.583	125.206
		Velocity (v_d)	mm/s	6.915	20.527	9.394	14.809	26.208
		Pressure (p)	mPa	21.461	8.180	29.774	10.812	29.648
		Power (W)	mW	0.03140	0.00598	0.06048	0.00974	0.06510
		ERP (W_{ERP})	mW	29.71800	25.32000	10.70000	8.43200	11.56600
		Radiation efficiency (σ)		0.00106	0.00024	0.00565	0.00115	0.00563
	wm	Frequency (ω)	Hz	12.371	42.818	48.207	85.598	113.245
		Velocity (v_d)	mm/s	6.263	18.616	8.511	13.481	23.486
		Pressure (p)	mPa	17.577	6.671	23.846	8.485	24.579
		Power (W)	mW	0.02107	0.00382	0.03878	0.00585	0.04365
		ERP (W_{ERP})	mW	24.34900	20.79900	8.75100	6.96500	9.35200
		Radiation efficiency (σ)		0.00087	0.00018	0.00443	0.00084	0.00467
	% reduction in velocity			9.43%	9.31%	9.40%	8.96%	10.39%
	% reduction in radiated pressure			18.10%	18.46%	19.91%	21.53%	17.10%
	% reduction in radiated power			32.92%	36.18%	35.87%	39.91%	32.94%
	% reduction in ERP			18.07%	17.86%	18.21%	17.40%	19.14%
	% reduction in Radiation efficiency			18.12%	22.31%	21.59%	27.25%	17.07%

wm: With surface modification; wom: Without surface modification;

Table 6: vibro-acoustic quantities at resonant frequencies for different base materials; boundary condition: CFCF.

		Mode no.	Unit	1	2	3	4	5
Steel base ($\sum m_d/m_p$ =0.079)	wom	Frequency (ω)	Hz	38.924	46.321	76.499	107.427	117.950
		Velocity (v_d)	mm/s	4.800	4.255	1.467	7.708	7.291
		Pressure (p)	mPa	21.400	6.978	2.527	1.649	1.365
		Power (W)	mW	0.03104	0.00348	0.00045	0.00217	0.00083
		ERP (W_{ERP})	mW	4.56800	2.13800	0.11200	2.46500	1.57800
		Radiation efficiency (σ)		0.00679	0.00163	0.00406	0.00088	0.00052
	wm	Frequency (ω)	Hz	37.459	44.628	73.743	103.387	113.631
		Velocity (v_d)	mm/s	4.614	4.099	1.419	7.395	7.012
		Pressure (p)	mPa	19.817	6.428	2.336	1.528	1.262
		Power (W)	mW	0.02663	0.00294	0.00039	0.00174	0.00066
		ERP (W_{ERP})	mW	4.22800	1.98100	0.10500	2.27900	1.45800
		Radiation efficiency (σ)		0.00630	0.00149	0.00370	0.00076	0.00045
	% reduction in velocity			3.87%	3.67%	3.26%	4.05%	3.82%
	% reduction in radiated pressure			7.40%	7.88%	7.57%	7.31%	7.54%
	% reduction in radiated power			14.22%	15.34%	14.70%	19.86%	20.60%
	% reduction in ERP			7.44%	7.33%	6.42%	7.53%	7.63%
	% reduction in Radiation efficiency			7.33%	8.65%	8.86%	13.33%	14.04%
Titanium base ($\sum m_d/m_p$ =0.137)	wom	Frequency (ω)	Hz	43.873	52.038	85.886	121.105	132.740
		Velocity (v_d)	mm/s	7.448	6.587	2.234	11.971	11.281
		Pressure (p)	mPa	37.289	12.347	4.421	2.871	2.386
		Power (W)	mW	0.09412	0.01098	0.00140	0.00817	0.00317
		ERP (W_{ERP})	mW	10.93100	5.14600	0.26400	5.90400	3.79600
		Radiation efficiency (σ)		0.00861	0.00213	0.00531	0.00138	0.00083
	wm	Frequency (ω)	Hz	41.110	48.852	80.700	113.488	124.602
		Velocity (v_d)	mm/s	6.965	6.182	2.112	11.161	10.558
		Pressure (p)	mPa	32.734	10.744	3.869	2.524	2.089
		Power (W)	mW	0.07258	0.00828	0.00107	0.00561	0.00213
		ERP (W_{ERP})	mW	9.58900	4.52300	0.23500	5.17000	3.31700
		Radiation efficiency (σ)		0.00757	0.00183	0.00454	0.00108	0.00064
	% reduction in velocity			6.47%	6.16%	5.50%	6.76%	6.40%
	% reduction in radiated pressure			12.21%	12.98%	12.48%	12.09%	12.44%
	% reduction in radiated power			22.88%	24.64%	23.65%	31.36%	32.64%
	% reduction in ERP			12.28%	12.12%	10.69%	12.42%	12.61%
	% reduction in Radiation efficiency			12.09%	14.25%	14.51%	21.63%	22.92%
Aluminium base ($\sum m_d/m_p$ =0.229)	wom	Frequency (ω)	Hz	44.493	52.506	86.588	122.848	134.291
		Velocity (v_d)	mm/s	12.331	10.871	3.598	19.849	18.606
		Pressure (p)	mPa	62.249	21.110	7.432	4.788	4.005
		Power (W)	mW	0.26223	0.03202	0.00394	0.02336	0.00932
		ERP (W_{ERP})	mW	29.68400	14.11200	0.69900	16.05500	10.39500
		Radiation efficiency (σ)		0.00883	0.00227	0.00564	0.00145	0.00090
	wm	Frequency (ω)	Hz	40.089	47.446	78.345	110.704	121.338
		Velocity (v_d)	mm/s	11.077	9.816	3.284	17.744	16.727
		Pressure (p)	mPa	50.523	16.897	6.004	3.894	3.237
		Power (W)	mW	0.17295	0.02037	0.00256	0.01278	0.00495
		ERP (W_{ERP})	mW	24.06500	11.46800	0.58200	12.98200	8.37200
		Radiation efficiency (σ)		0.00719	0.00178	0.00440	0.00098	0.00059
	% reduction in velocity			10.17%	9.71%	8.73%	10.60%	10.10%
	% reduction in radiated pressure			18.84%	19.95%	19.21%	18.67%	19.19%
	% reduction in radiated power			34.05%	36.40%	35.05%	45.27%	46.87%
	% reduction in ERP			18.93%	18.74%	16.68%	19.14%	19.46%
	% reduction in Radiation efficiency			18.65%	21.73%	22.04%	32.32%	34.03%

wm: With surface modification; wom: Without surface modification;

Table 7: vibro-acoustic quantities at resonant frequencies for different base materials; boundary condition: CFSF.

		Mode no.	Unit	1	2	3	4	5
Steel base ($\sum m_d/m_p$ =0.079)	wom	Frequency (ω)	Hz	26.742	36.096	69.645	86.959	98.872
		Velocity (v_d)	mm/s	3.510	3.172	2.312	6.866	6.343
		Pressure (p)	mPa	16.263	3.422	0.924	3.996	2.411
		Power (W)	mW	0.01797	0.00087	0.00009	0.00225	0.00068
		ERP (W_{ERP})	mW	5.11000	1.91400	0.18100	2.85400	1.55800
		Radiation efficiency (σ)		0.00352	0.00045	0.00048	0.00079	0.00043
	wm	Frequency (ω)	Hz	25.735	34.777	67.136	83.689	95.252
		Velocity (v_d)	mm/s	3.377	3.058	2.237	6.596	6.111
		Pressure (p)	mPa	15.060	3.159	0.850	3.700	2.223
		Power (W)	mW	0.01542	0.00073	0.00007	0.00186	0.00056
		ERP (W_{ERP})	mW	4.73100	1.77700	0.17000	2.64100	1.44300
		Radiation efficiency (σ)		0.00326	0.00041	0.00043	0.00070	0.00039
	% reduction in velocity			3.80%	3.61%	3.24%	3.92%	3.66%
	% reduction in radiated pressure			7.39%	7.67%	8.00%	7.40%	7.81%
	% reduction in radiated power			14.23%	15.16%	16.54%	17.48%	17.73%
	% reduction in ERP			7.41%	7.19%	6.24%	7.45%	7.39%
	% reduction in Radiation efficiency			7.37%	8.59%	10.99%	10.83%	11.17%
Titanium base ($\sum m_d/m_p$ =0.137)	wom	Frequency (ω)	Hz	30.128	40.468	78.150	98.017	111.192
		Velocity (v_d)	mm/s	5.437	4.902	3.521	10.658	9.804
		Pressure (p)	mPa	28.330	6.038	1.640	6.968	4.248
		Power (W)	mW	0.05451	0.00274	0.00029	0.00779	0.00235
		ERP (W_{ERP})	mW	12.22300	4.60500	0.41900	6.83300	3.74400
		Radiation efficiency (σ)		0.00446	0.00060	0.00069	0.00114	0.00063
	wm	Frequency (ω)	Hz	28.230	37.990	73.431	91.852	104.376
		Velocity (v_d)	mm/s	5.091	4.605	3.328	9.959	9.202
		Pressure (p)	mPa	24.872	5.275	1.425	6.116	3.701
		Power (W)	mW	0.04203	0.00207	0.00021	0.00559	0.00168
		ERP (W_{ERP})	mW	10.72800	4.05700	0.37500	5.99300	3.28600
		Radiation efficiency (σ)		0.00392	0.00051	0.00057	0.00093	0.00051
	% reduction in velocity			6.36%	6.05%	5.48%	6.56%	6.14%
	% reduction in radiated pressure			12.21%	12.65%	13.14%	12.23%	12.87%
	% reduction in radiated power			22.90%	24.42%	26.52%	28.23%	28.71%
	% reduction in ERP			12.23%	11.90%	10.43%	12.30%	12.21%
	% reduction in Radiation efficiency			12.16%	14.22%	17.96%	18.17%	18.79%
Aluminium base ($\sum m_d/m_p$ =0.229)	wom	Frequency (ω)	Hz	30.530	40.700	78.724	99.405	112.369
		Velocity (v_d)	mm/s	8.975	8.065	5.669	17.660	16.141
		Pressure (p)	mPa	47.271	10.281	2.816	11.642	7.215
		Power (W)	mW	0.15174	0.00792	0.00083	0.02207	0.00687
		ERP (W_{ERP})	mW	33.17100	12.61000	1.08400	18.56900	10.23100
		Radiation efficiency (σ)		0.00457	0.00063	0.00076	0.00119	0.00067
	wm	Frequency (ω)	Hz	27.507	36.778	71.230	89.580	101.532
		Velocity (v_d)	mm/s	8.079	7.296	5.175	15.840	14.576
		Pressure (p)	mPa	38.371	8.279	2.250	9.447	5.785
		Power (W)	mW	0.10004	0.00506	0.00051	0.01298	0.00400
		ERP (W_{ERP})	mW	26.91600	10.29100	0.90700	15.04800	8.30000
		Radiation efficiency (σ)		0.00372	0.00049	0.00056	0.00086	0.00048
	% reduction in velocity			9.99%	9.54%	8.72%	10.30%	9.70%
	% reduction in radiated pressure			18.83%	19.48%	20.10%	18.86%	19.81%
	% reduction in radiated power			34.07%	36.08%	38.71%	41.19%	41.78%
	% reduction in ERP			18.86%	18.39%	16.37%	18.96%	18.88%
	% reduction in Radiation efficiency			18.75%	21.67%	26.71%	27.43%	28.24%

wm: With surface modification; wom: Without surface modification;

Table 8: vibro-acoustic quantities at resonant frequencies for different base materials; boundary condition: CFFF.

		Mode no.	Unit	1	2	3	4	5
Steel base ($\sum m_d/m_p$ =0.079)	wom	Frequency (ω)	Hz	6.109	14.897	37.517	47.883	54.470
		Velocity (v_d)	mm/s	2.355	6.814	10.449	6.184	14.210
		Pressure (p)	mPa	4.044	0.276	11.302	3.169	1.642
		Power (W)	mW	0.00112	0.00001	0.00870	0.00070	0.00028
		ERP (W_{ERP})	mW	7.25400	4.68100	5.11800	0.88400	2.77800
		Radiation efficiency (σ)		0.00015	0.00000	0.00170	0.00079	0.00010
	wm	Frequency (ω)	Hz	5.886	14.366	36.158	46.190	52.542
		Velocity (v_d)	mm/s	2.269	6.574	10.044	5.988	13.685
		Pressure (p)	mPa	3.754	0.256	10.453	2.918	1.520
		Power (W)	mW	0.00096	0.00001	0.00744	0.00059	0.00023
		ERP (W_{ERP})	mW	6.73600	4.35600	4.74200	0.82700	2.58100
		Radiation efficiency (σ)		0.00014	0.00000	0.00157	0.00072	0.00009
	% reduction in velocity			3.64%	3.52%	3.88%	3.18%	3.69%
	% reduction in radiated pressure			7.16%	7.21%	7.51%	7.93%	7.45%
	% reduction in radiated power			13.80%	16.66%	14.45%	15.29%	16.36%
	% reduction in ERP			7.15%	6.95%	7.34%	6.46%	7.10%
	% reduction in Radiation efficiency			7.16%	10.43%	7.67%	9.44%	9.96%
Titanium base ($\sum m_d/m_p$ =0.137)	wom	Frequency (ω)	Hz	6.885	16.627	42.150	53.890	61.005
		Velocity (v_d)	mm/s	3.605	10.524	16.462	9.739	22.195
		Pressure (p)	mPa	6.991	0.482	19.766	5.511	2.873
		Power (W)	mW	0.00333	0.00003	0.02660	0.00213	0.00092
		ERP (W_{ERP})	mW	17.08100	11.28200	12.46500	2.09100	6.74900
		Radiation efficiency (σ)		0.00020	0.00000	0.00213	0.00102	0.00014
	wm	Frequency (ω)	Hz	6.464	15.634	39.593	50.693	57.385
		Velocity (v_d)	mm/s	3.385	9.902	15.398	9.211	20.825
		Pressure (p)	mPa	6.163	0.425	17.324	4.797	2.518
		Power (W)	mW	0.00259	0.00003	0.02043	0.00161	0.00068
		ERP (W_{ERP})	mW	15.06000	9.98300	10.95800	1.86600	5.95600
		Radiation efficiency (σ)		0.00017	0.00000	0.00186	0.00086	0.00011
	% reduction in velocity			6.10%	5.90%	6.47%	5.43%	6.17%
	% reduction in radiated pressure			11.84%	11.93%	12.35%	12.96%	12.37%
	% reduction in radiated power			22.28%	26.98%	23.17%	24.35%	26.61%
	% reduction in ERP			11.83%	11.51%	12.09%	10.77%	11.75%
	% reduction in Radiation efficiency			11.85%	17.48%	12.60%	15.21%	16.84%
Aluminium base ($\sum m_d/m_p$ =0.229)	wom	Frequency (ω)	Hz	6.980	16.608	42.517	54.537	61.260
		Velocity (v_d)	mm/s	5.846	17.290	27.826	16.655	37.104
		Pressure (p)	mPa	11.529	0.807	33.117	9.130	4.843
		Power (W)	mW	0.00907	0.00010	0.07465	0.00584	0.00262
		ERP (W_{ERP})	mW	45.22100	30.96000	34.66800	5.65100	18.75100
		Radiation efficiency (σ)		0.00020	0.00000	0.00215	0.00103	0.00014
	wm	Frequency (ω)	Hz	6.308	15.045	38.459	49.432	55.529
		Velocity (v_d)	mm/s	5.284	15.680	25.015	15.193	33.506
		Pressure (p)	mPa	9.418	0.658	26.837	7.331	3.912
		Power (W)	mW	0.00605	0.00006	0.04903	0.00376	0.00160
		ERP (W_{ERP})	mW	36.94800	25.43900	28.22200	4.69700	15.34600
		Radiation efficiency (σ)		0.00016	0.00000	0.00174	0.00080	0.00010
	% reduction in velocity			9.60%	9.31%	10.10%	8.78%	9.70%
	% reduction in radiated pressure			18.31%	18.44%	18.96%	19.70%	19.22%
	% reduction in radiated power			33.27%	39.47%	34.32%	35.69%	39.15%
	% reduction in ERP			18.29%	17.83%	18.60%	16.89%	18.16%
	% reduction in Radiation efficiency			18.33%	26.33%	19.32%	22.62%	25.64%

wm: With surface modification; wom: Without surface modification;

Table 9: vibro-acoustic quantities at resonant frequencies for different base materials; boundary condition: CSCG.

		Mode no.	Unit	1	2	3	4	5
Steel base ($\sum m_d/m_p$ =0.079)	wom	Frequency (ω)	Hz	41.570	68.271	110.905	132.544	138.865
		Velocity (v_d)	mm/s	3.039	4.981	4.861	6.140	8.399
		Pressure (p)	mPa	16.174	9.672	2.681	1.923	1.691
		Power (W)	mW	0.01773	0.00672	0.00175	0.00053	0.00111
		ERP (W_{ERP})	mW	1.36200	1.46600	0.78700	0.69500	1.27900
		Radiation efficiency (σ)		0.01301	0.00458	0.00222	0.00077	0.00086
	wm	Frequency (ω)	Hz	40.000	65.693	106.716	127.539	133.621
		Velocity (v_d)	mm/s	2.930	4.783	4.696	5.885	8.053
		Pressure (p)	mPa	14.997	8.895	2.476	1.771	1.552
		Power (W)	mW	0.01525	0.00566	0.00143	0.00043	0.00087
		ERP (W_{ERP})	mW	1.26400	1.35400	0.73300	0.64200	1.18000
		Radiation efficiency (σ)		0.01206	0.00418	0.00194	0.00067	0.00074
	% reduction in velocity			3.60%	3.97%	3.40%	4.15%	4.11%
	% reduction in radiated pressure			7.28%	8.03%	7.67%	7.88%	8.22%
	% reduction in radiated power			14.00%	15.69%	18.44%	18.86%	21.12%
	% reduction in ERP			7.18%	7.62%	6.86%	7.65%	7.74%
	% reduction in Radiation efficiency			7.34%	8.74%	12.43%	12.14%	14.50%
Titanium base ($\sum m_d/m_p$ =0.137)	wom	Frequency (ω)	Hz	46.879	76.990	125.068	149.471	156.598
		Velocity (v_d)	mm/s	4.695	7.695	7.510	9.485	12.975
		Pressure (p)	mPa	28.177	16.852	4.671	3.349	2.945
		Power (W)	mW	0.05374	0.02070	0.00631	0.00188	0.00418
		ERP (W_{ERP})	mW	3.25100	3.49900	1.87900	1.65900	3.05200
		Radiation efficiency (σ)		0.01653	0.00592	0.00336	0.00114	0.00137
	wm	Frequency (ω)	Hz	43.917	72.126	117.165	140.028	146.705
		Velocity (v_d)	mm/s	4.412	7.185	7.082	8.828	12.085
		Pressure (p)	mPa	24.787	14.627	4.081	2.915	2.547
		Power (W)	mW	0.04162	0.01549	0.00445	0.00131	0.00278
		ERP (W_{ERP})	mW	2.86500	3.05900	1.66500	1.45000	2.66300
		Radiation efficiency (σ)		0.01453	0.00506	0.00267	0.00090	0.00104
	% reduction in velocity			6.03%	6.63%	5.71%	6.92%	6.86%
	% reduction in radiated pressure			12.03%	13.20%	12.64%	12.97%	13.51%
	% reduction in radiated power			22.56%	25.19%	29.49%	30.50%	33.43%
	% reduction in ERP			11.88%	12.56%	11.38%	12.61%	12.75%
	% reduction in Radiation efficiency			12.11%	14.44%	20.43%	20.47%	23.70%
Aluminium base ($\sum m_d/m_p$ =0.229)	wom	Frequency (ω)	Hz	47.581	78.143	126.940	151.708	158.942
		Velocity (v_d)	mm/s	7.720	12.653	12.349	15.595	21.334
		Pressure (p)	mPa	47.024	28.124	7.796	5.589	4.915
		Power (W)	mW	0.14964	0.05779	0.01796	0.00536	0.01197
		ERP (W_{ERP})	mW	8.79000	9.45900	5.07900	4.48500	8.25200
		Radiation efficiency (σ)		0.01702	0.00611	0.00353	0.00119	0.00145
	wm	Frequency (ω)	Hz	42.854	70.381	114.330	136.643	143.158
		Velocity (v_d)	mm/s	6.986	11.338	11.235	13.907	19.045
		Pressure (p)	mPa	38.287	22.429	6.280	4.476	3.898
		Power (W)	mW	0.09931	0.03634	0.01025	0.00299	0.00624
		ERP (W_{ERP})	mW	7.17500	7.63000	4.18200	3.61500	6.63400
		Radiation efficiency (σ)		0.01384	0.00476	0.00245	0.00083	0.00094
	% reduction in velocity			9.51%	10.39%	9.02%	10.83%	10.73%
	% reduction in radiated pressure			18.58%	20.25%	19.45%	19.92%	20.69%
	% reduction in radiated power			33.63%	37.12%	42.90%	44.09%	47.82%
	% reduction in ERP			18.37%	19.33%	17.66%	19.40%	19.61%
	% reduction in Radiation efficiency			18.69%	22.04%	30.66%	30.62%	35.09%

wm: With surface modification; wom: Without surface modification;

Table 10: vibro-acoustic quantities at resonant frequencies for different base materials; boundary condition: CCCC.

		Mode no.	Unit	1	2	3	4	5
Steel base ($\sum m_d/m_p$ =0.079)	wom	Frequency (ω)	Hz	62.849	128.226	128.229	189.180	230.012
		Velocity (v_d)	mm/s	0.449	1.395	1.395	1.510	1.744
		Pressure (p)	mPa	6.357	0.244	0.244	2.006	13.177
		Power (W)	mW	0.00273	0.00054	0.00054	0.00028	0.00875
		ERP (W_{ERP})	mW	0.10700	0.20600	0.20600	0.14300	0.14900
		Radiation efficiency (σ)		0.02555	0.00261	0.00261	0.00194	0.05879
	wm	Frequency (ω)	Hz	60.434	123.299	123.303	181.913	221.176
		Velocity (v_d)	mm/s	0.432	1.342	1.342	1.452	1.677
		Pressure (p)	mPa	5.877	0.226	0.226	1.855	12.185
		Power (W)	mW	0.00233	0.00043	0.00043	0.00023	0.00765
		ERP (W_{ERP})	mW	0.09900	0.19100	0.19100	0.13200	0.13800
		Radiation efficiency (σ)		0.02364	0.00223	0.00223	0.00177	0.05564
	% reduction in velocity			3.84%	3.84%	3.84%	3.84%	3.84%
	% reduction in radiated pressure			7.54%	7.47%	7.47%	7.53%	7.53%
	% reduction in radiated power			14.46%	20.70%	20.70%	15.71%	12.50%
	% reduction in ERP			7.54%	7.54%	7.54%	7.54%	7.53%
	% reduction in Radiation efficiency			7.48%	14.24%	14.24%	8.84%	5.37%
Titanium base ($\sum m_d/m_p$ =0.137)	wom	Frequency (ω)	Hz	70.876	144.602	144.605	213.340	259.386
		Velocity (v_d)	mm/s	0.693	2.156	2.155	2.333	2.694
		Pressure (p)	mPa	11.074	0.424	0.424	3.493	22.952
		Power (W)	mW	0.00826	0.00205	0.00205	0.00090	0.02441
		ERP (W_{ERP})	mW	0.25500	0.49200	0.49200	0.34200	0.35500
		Radiation efficiency (σ)		0.03242	0.00417	0.00417	0.00263	0.06877
	wm	Frequency (ω)	Hz	66.322	135.314	135.317	199.638	242.728
		Velocity (v_d)	mm/s	0.649	2.017	2.017	2.183	2.521
		Pressure (p)	mPa	9.697	0.372	0.372	3.059	20.100
		Power (W)	mW	0.00634	0.00139	0.00139	0.00066	0.01965
		ERP (W_{ERP})	mW	0.22300	0.43100	0.43100	0.29900	0.31100
		Radiation efficiency (σ)		0.02842	0.00322	0.00322	0.00221	0.06322
	% reduction in velocity			6.42%	6.42%	6.42%	6.42%	6.42%
	% reduction in radiated pressure			12.43%	12.32%	12.32%	12.42%	12.42%
	% reduction in radiated power			23.23%	32.45%	32.45%	26.39%	19.50%
	% reduction in ERP			12.44%	12.43%	12.43%	12.43%	12.43%
	% reduction in Radiation efficiency			12.33%	22.86%	22.86%	15.94%	8.07%
Aluminium base ($\sum m_d/m_p$ =0.229)	wom	Frequency (ω)	Hz	71.936	146.766	146.769	216.532	263.267
		Velocity (v_d)	mm/s	1.140	3.545	3.544	3.836	4.430
		Pressure (p)	mPa	18.481	0.708	0.708	5.829	38.303
		Power (W)	mW	0.0230	0.0059	0.0059	0.0025	0.0672
		ERP (W_{ERP})	mW	0.6890	1.3310	1.3310	0.9240	0.9600
		Radiation efficiency (σ)		0.0334	0.0044	0.0044	0.0027	0.0700
	wm	Frequency (ω)	Hz	64.679	131.961	131.964	194.692	236.715
		Velocity (v_d)	mm/s	1.025	3.187	3.186	3.449	3.983
		Pressure (p)	mPa	14.940	0.574	0.574	4.714	30.971
		Power (W)	mW	0.0151	0.0031	0.0031	0.0016	0.0475
		ERP (W_{ERP})	mW	0.5570	1.0760	1.0760	0.7470	0.7760
		Radiation efficiency (σ)		0.0270	0.0029	0.0029	0.0021	0.0611
	% reduction in velocity			10.09%	10.09%	10.09%	10.09%	10.09%
	% reduction in radiated pressure			19.16%	18.99%	18.99%	19.13%	19.14%
	% reduction in radiated power			34.52%	46.66%	46.66%	38.70%	29.39%
	% reduction in ERP			19.16%	19.16%	19.16%	19.15%	19.15%
	% reduction in Radiation efficiency			19.00%	34.02%	34.02%	24.18%	12.66%

wm: With surface modification; wom: Without surface modification;

Table 11: Vibro-acoustic quantities at the first five resonant frequencies for different array spacings and different plate materials under SSSS and SCSC boundary conditions.

			SSSS					SCSC				
			Mode no.					Mode no.				
Material	Spacing	Parameters	1	2	3	4	5	1	2	3	4	5
Steel	-	Frequency (Hz)	34.454	86.136	86.136	137.817	172.271	50.543	95.641	121.018	165.209	178.648
		Velocity (mm/s)	5.884	12.227	12.227	10.410	6.903	4.010	7.079	0.486	3.937	6.977
		Pressure (mPa)	23.165	1.889	1.889	2.130	24.998	21.356	2.479	1.531	6.079	18.106
	1 mm	Frequency (Hz)	33.130	82.826	82.826	132.523	165.653	48.601	91.967	116.368	158.862	171.786
		Velocity (mm/s)	5.658	11.757	11.757	10.010	6.638	3.856	6.807	0.468	3.785	6.709
		Pressure (mPa)	21.419	1.747	1.747	1.970	23.117	19.746	2.293	1.416	5.622	16.744
	2 mm	Frequency (Hz)	34.108	85.271	85.271	136.434	170.542	50.036	94.682	119.803	163.551	176.856
		Velocity (mm/s)	5.825	12.105	12.105	10.305	6.834	3.970	7.007	0.481	3.897	6.907
		Pressure (mPa)	22.703	1.851	1.851	2.088	24.500	20.929	2.430	1.501	5.958	17.745
	5 mm	Frequency (Hz)	34.398	85.995	85.995	137.593	171.991	50.461	95.486	120.821	164.940	178.358
		Velocity (mm/s)	5.875	12.207	12.207	10.393	6.892	4.003	7.067	0.485	3.930	6.966
		Pressure (mPa)	23.090	1.883	1.883	2.123	24.917	21.286	2.471	1.526	6.059	18.047
Titanium	-	Frequency (Hz)	38.854	97.136	97.136	155.419	194.273	56.998	107.856	136.474	186.309	201.464
		Velocity (mm/s)	9.090	18.889	18.889	16.081	10.664	6.194	10.935	0.751	6.082	10.779
		Pressure (mPa)	40.356	3.290	3.290	3.705	43.537	37.204	4.319	2.666	10.581	31.530
	1 mm	Frequency (Hz)	36.358	90.897	90.897	145.437	181.796	53.336	100.928	127.707	174.343	188.526
		Velocity (mm/s)	8.506	17.676	17.676	15.048	9.979	5.796	10.233	0.703	5.691	10.086
		Pressure (mPa)	35.338	2.881	2.881	3.247	38.131	32.578	3.782	2.335	9.269	27.617
	2 mm	Frequency (Hz)	38.182	95.457	95.457	152.732	190.914	56.012	105.991	134.114	183.087	197.981
		Velocity (mm/s)	8.933	18.562	18.562	15.803	10.480	6.087	10.746	0.738	5.976	10.592
		Pressure (mPa)	38.973	3.178	3.178	3.579	42.047	35.929	4.171	2.575	10.219	30.452
	5 mm	Frequency (Hz)	38.744	96.862	96.862	154.979	193.723	56.837	107.551	136.088	185.782	200.894
		Velocity (mm/s)	9.065	18.836	18.836	16.035	10.634	6.177	10.904	0.749	6.064	10.748
		Pressure (mPa)	40.128	3.272	3.272	3.684	43.292	36.994	4.295	2.651	10.521	31.353
Aluminium	-	Frequency (Hz)	39.436	98.590	98.590	157.745	197.180	57.851	109.470	138.516	189.097	204.479
		Velocity (mm/s)	14.947	31.059	31.059	26.442	17.535	10.185	17.980	1.235	10.000	17.723
		Pressure (mPa)	67.351	5.491	5.491	6.182	72.656	62.090	7.208	4.449	17.656	52.618
	1 mm	Frequency (Hz)	35.457	88.645	88.645	141.834	177.293	52.014	98.427	124.543	170.023	183.856
		Velocity (mm/s)	13.439	27.926	27.926	23.774	15.766	9.158	16.166	1.111	8.991	15.936
		Pressure (mPa)	54.446	4.440	4.440	5.005	58.755	50.193	5.827	3.598	14.283	42.555
	2 mm	Frequency (Hz)	38.317	95.793	95.793	153.270	191.588	56.210	106.365	134.587	183.733	198.680
		Velocity (mm/s)	14.523	30.178	30.178	25.692	17.038	9.896	17.470	1.200	9.716	17.220
		Pressure (mPa)	63.583	5.184	5.184	5.839	68.598	58.616	6.805	4.200	16.671	49.681
	5 mm	Frequency (Hz)	39.250	98.126	98.126	157.002	196.252	57.579	108.955	137.864	188.207	203.517
		Velocity (mm/s)	14.877	30.913	30.913	26.317	17.452	10.137	17.896	1.229	9.953	17.640
		Pressure (mPa)	66.718	5.440	5.440	6.125	71.975	61.507	7.140	4.407	17.491	52.125

Table 12: Vibro-acoustic quantities at the first five resonant frequencies for different array spacings and different plate materials under CCFF and CFCF boundary conditions.

			CCFF					CFCF				
			Mode no.					Mode no.				
Material	Spacing	Parameters	1	2	3	4	5	1	2	3	4	5
Steel	-	Frequency (Hz)	12.126	42.005	46.661	83.774	110.182	38.924	46.318	76.492	107.417	117.937
		Velocity (mm/s)	2.746	8.135	3.930	5.877	10.522	4.800	4.255	1.466	7.709	7.290
		Pressure (mPa)	7.487	3.183	9.940	3.570	11.255	21.400	6.980	2.528	1.649	1.365
	1 mm	Frequency (Hz)	11.687	40.501	44.954	80.759	106.189	37.458	44.624	73.736	103.377	113.618
		Velocity (mm/s)	2.648	7.849	3.797	5.677	10.107	4.614	4.099	1.419	7.397	7.011
		Pressure (mPa)	6.958	2.955	9.156	3.263	10.507	19.817	6.431	2.336	1.529	1.262
	2 mm	Frequency (Hz)	12.011	41.610	46.213	82.982	109.135	38.541	45.874	75.769	106.360	116.805
		Velocity (mm/s)	2.720	8.059	3.895	5.824	10.414	4.752	4.214	1.454	7.627	7.217
		Pressure (mPa)	7.346	3.122	9.733	3.489	11.055	20.980	6.835	2.477	1.617	1.338
	5 mm	Frequency (Hz)	12.107	41.939	46.587	83.643	110.009	38.861	46.245	76.373	107.244	117.751
		Velocity (mm/s)	2.742	8.122	3.924	5.868	10.505	4.792	4.248	1.464	7.696	7.278
		Pressure (mPa)	7.464	3.173	9.907	3.557	11.221	21.331	6.957	2.520	1.644	1.360
Titanium	-	Frequency (Hz)	13.596	47.062	52.598	93.962	123.900	43.873	52.035	85.880	121.095	132.727
		Velocity (mm/s)	4.228	12.531	5.907	9.054	16.118	7.448	6.587	2.234	11.972	11.280
		Pressure (mPa)	12.971	5.273	17.519	6.321	18.850	37.289	12.350	4.422	2.871	2.386
	1 mm	Frequency (Hz)	12.771	44.238	49.377	88.295	116.380	41.110	48.849	80.693	113.478	124.589
		Velocity (mm/s)	3.975	11.793	5.564	8.538	15.053	6.965	6.181	2.111	11.163	10.558
		Pressure (mPa)	11.454	4.645	15.246	5.430	16.773	32.734	10.748	3.870	2.524	2.089
	2 mm	Frequency (Hz)	13.374	46.299	51.729	92.432	121.871	43.129	51.175	84.479	119.043	130.532
		Velocity (mm/s)	4.160	12.332	5.814	8.914	15.832	7.318	6.477	2.201	11.755	11.086
		Pressure (mPa)	12.553	5.099	16.893	6.076	18.275	36.033	11.909	4.270	2.775	2.304
	5 mm	Frequency (Hz)	13.559	46.934	52.454	93.706	123.561	43.751	51.892	85.646	120.757	132.362
		Velocity (mm/s)	4.217	12.498	5.891	9.030	16.072	7.426	6.569	2.228	11.937	11.248
		Pressure (mPa)	12.901	5.244	17.417	6.282	18.751	37.081	12.279	4.397	2.855	2.373
Aluminium	-	Frequency (Hz)	13.678	47.286	53.343	94.573	125.193	44.493	52.503	86.582	122.838	134.278
		Velocity (mm/s)	6.915	20.531	9.395	14.817	26.202	12.331	10.870	3.598	19.851	18.605
		Pressure (mPa)	21.461	8.175	29.762	10.814	29.610	62.249	21.116	7.433	4.789	4.006
	1 mm	Frequency (Hz)	12.371	42.815	48.201	85.588	113.232	40.089	47.443	78.339	110.694	121.325
		Velocity (mm/s)	6.263	18.620	8.512	13.489	23.480	11.077	9.815	3.283	17.747	16.726
		Pressure (mPa)	17.577	6.665	23.835	8.485	24.544	50.523	16.903	6.005	3.895	3.238
	2 mm	Frequency (Hz)	13.310	46.028	51.896	92.045	121.829	43.254	51.079	84.262	119.423	130.635
		Velocity (mm/s)	6.732	19.994	9.146	14.443	25.437	11.978	10.573	3.509	19.260	18.076
		Pressure (mPa)	20.329	7.735	28.030	10.131	28.136	58.828	19.884	7.016	4.528	3.782
	5 mm	Frequency (Hz)	13.615	47.073	53.099	94.145	124.624	44.286	52.263	86.189	122.267	133.664
		Velocity (mm/s)	6.884	20.440	9.353	14.752	26.076	12.272	10.821	3.582	19.755	18.517
		Pressure (mPa)	21.267	8.100	29.475	10.703	29.352	61.671	20.912	7.363	4.745	3.969

Table 13: Vibro-acoustic quantities at the first five resonant frequencies for different array spacings and different plate materials under CFSF and CFFF boundary conditions.

			CFSF					CFFF				
			Mode no.					Mode no.				
Material	Spacing	Parameters	1	2	3	4	5	1	2	3	4	5
Steel	-	Frequency (Hz)	26.742	36.093	69.639	86.949	98.858	6.109	14.893	37.510	47.873	54.457
		Velocity (mm/s)	3.510	3.173	2.311	6.868	6.342	2.355	6.818	10.458	6.180	14.212
		Pressure (mPa)	16.263	3.423	0.925	3.993	2.413	4.044	0.276	11.296	3.176	1.643
	1 mm	Frequency (Hz)	25.735	34.773	67.130	83.679	95.238	5.886	14.363	36.152	46.180	52.529
		Velocity (mm/s)	3.377	3.058	2.236	6.599	6.110	2.269	6.579	10.052	5.984	13.688
		Pressure (mPa)	15.060	3.160	0.851	3.697	2.225	3.754	0.256	10.448	2.925	1.520
	2 mm	Frequency (Hz)	26.479	35.747	68.980	86.094	97.909	6.051	14.754	37.154	47.429	53.951
		Velocity (mm/s)	3.475	3.143	2.291	6.798	6.281	2.333	6.755	10.352	6.128	14.075
		Pressure (mPa)	15.944	3.353	0.905	3.914	2.363	3.967	0.271	11.071	3.110	1.610
	5 mm	Frequency (Hz)	26.699	36.036	69.530	86.809	98.702	6.100	14.870	37.452	47.799	54.373
		Velocity (mm/s)	3.505	3.168	2.308	6.857	6.332	2.351	6.808	10.441	6.171	14.189
		Pressure (mPa)	16.211	3.411	0.922	3.980	2.405	4.031	0.275	11.260	3.166	1.637
Titanium	-	Frequency (Hz)	30.128	40.464	78.143	98.007	111.179	6.885	16.623	42.143	53.880	60.992
		Velocity (mm/s)	5.437	4.903	3.520	10.661	9.802	3.605	10.530	16.474	9.732	22.198
		Pressure (mPa)	28.330	6.040	1.641	6.963	4.250	6.991	0.482	19.757	5.522	2.874
	1 mm	Frequency (Hz)	28.230	37.987	73.424	91.843	104.363	6.464	15.631	39.587	50.683	57.372
		Velocity (mm/s)	5.091	4.606	3.327	9.962	9.200	3.385	9.909	15.409	9.204	20.828
		Pressure (mPa)	24.872	5.276	1.426	6.111	3.703	6.163	0.425	17.316	4.808	2.518
	2 mm	Frequency (Hz)	29.617	39.796	76.869	96.346	109.340	6.771	16.355	41.453	53.016	60.014
		Velocity (mm/s)	5.344	4.822	3.467	10.473	9.640	3.546	10.362	16.187	9.588	21.828
		Pressure (mPa)	27.376	5.829	1.582	6.728	4.100	6.763	0.467	19.084	5.326	2.775
	5 mm	Frequency (Hz)	30.044	40.353	77.931	97.734	110.873	6.866	16.578	42.028	53.735	60.827
		Velocity (mm/s)	5.421	4.889	3.511	10.631	9.775	3.595	10.502	16.427	9.707	22.137
		Pressure (mPa)	28.172	6.005	1.632	6.924	4.226	6.953	0.480	19.646	5.490	2.857
Aluminium	-	Frequency (Hz)	30.530	40.696	78.718	99.395	112.356	6.980	16.605	42.510	54.527	61.246
		Velocity (mm/s)	8.975	8.066	5.668	17.665	16.138	5.846	17.300	27.845	16.640	37.110
		Pressure (mPa)	47.271	10.284	2.818	11.634	7.219	11.529	0.808	33.102	9.148	4.844
	1 mm	Frequency (Hz)	27.507	36.774	71.224	89.570	101.519	6.308	15.041	38.452	49.422	55.516
		Velocity (mm/s)	8.079	7.297	5.173	15.846	14.573	5.284	15.690	25.035	15.178	33.511
		Pressure (mPa)	38.371	8.281	2.252	9.440	5.789	9.418	0.659	26.824	7.347	3.913
	2 mm	Frequency (Hz)	29.680	39.593	76.609	96.632	109.308	6.791	16.165	41.369	53.090	59.634
		Velocity (mm/s)	8.723	7.850	5.528	17.153	15.697	5.688	16.847	27.055	16.228	36.098
		Pressure (mPa)	44.675	9.698	2.652	10.994	6.801	10.914	0.764	31.271	8.623	4.572
	5 mm	Frequency (Hz)	30.388	40.510	78.361	98.933	111.842	6.948	16.530	42.317	54.282	60.972
		Velocity (mm/s)	8.933	8.029	5.643	17.580	16.064	5.819	17.223	27.714	16.568	36.940
		Pressure (mPa)	46.833	10.186	2.791	11.526	7.150	11.424	0.800	32.794	9.061	4.798

Table 14: Vibro-acoustic quantities at the first five resonant frequencies for different array spacings and different plate materials under CSCG and CCCC boundary conditions.

			CSCG					CCCC				
			Mode no.					Mode no.				
Material	Spacing	Parameters	1	2	3	4	5	1	2	3	4	5
Steel	-	Frequency (Hz)	41.570	68.268	110.898	132.534	138.852	62.849	128.223	128.223	189.170	229.998
		Velocity (mm/s)	3.039	4.982	4.863	6.135	8.399	0.449	1.396	1.396	1.510	1.744
		Pressure (mPa)	16.174	9.669	2.682	1.923	1.692	6.357	0.244	0.244	2.005	13.174
	1 mm	Frequency (Hz)	40.000	65.690	106.709	127.529	133.608	60.434	123.296	123.296	181.903	221.163
		Velocity (mm/s)	2.930	4.784	4.698	5.880	8.054	0.432	1.342	1.342	1.452	1.677
		Pressure (mPa)	14.997	8.892	2.476	1.772	1.553	5.877	0.226	0.226	1.854	12.182
	2 mm	Frequency (Hz)	41.160	67.594	109.803	131.225	137.480	62.218	126.936	126.936	187.272	227.691
		Velocity (mm/s)	3.010	4.930	4.819	6.069	8.309	0.444	1.382	1.382	1.495	1.727
		Pressure (mPa)	15.861	9.464	2.627	1.883	1.655	6.230	0.239	0.239	1.965	12.911
	5 mm	Frequency (Hz)	41.503	68.158	110.720	132.321	138.628	62.747	128.014	128.014	188.862	229.624
		Velocity (mm/s)	3.034	4.974	4.856	6.125	8.385	0.448	1.393	1.393	1.508	1.741
		Pressure (mPa)	16.122	9.636	2.673	1.917	1.686	6.336	0.243	0.243	1.998	13.131
Titanium	-	Frequency (Hz)	46.879	76.987	125.062	149.461	156.585	70.876	144.599	144.599	213.330	259.372
		Velocity (mm/s)	4.695	7.696	7.512	9.478	12.975	0.693	2.156	2.156	2.333	2.694
		Pressure (mPa)	28.177	16.847	4.672	3.350	2.946	11.074	0.425	0.425	3.492	22.946
	1 mm	Frequency (Hz)	43.917	72.122	117.158	140.018	146.692	66.322	135.310	135.310	199.628	242.715
		Velocity (mm/s)	4.412	7.186	7.084	8.822	12.085	0.649	2.017	2.017	2.183	2.521
		Pressure (mPa)	24.787	14.623	4.081	2.915	2.548	9.697	0.372	0.372	3.058	20.095
	2 mm	Frequency (Hz)	46.081	75.676	122.933	146.917	153.920	69.650	142.098	142.098	209.641	254.889
		Velocity (mm/s)	4.618	7.559	7.396	9.302	12.736	0.681	2.119	2.119	2.293	2.648
		Pressure (mPa)	27.242	16.234	4.509	3.230	2.836	10.694	0.410	0.410	3.372	22.160
	5 mm	Frequency (Hz)	46.748	76.771	124.711	149.043	156.147	70.675	144.190	144.190	212.726	258.639
		Velocity (mm/s)	4.682	7.674	7.493	9.450	12.937	0.691	2.150	2.150	2.327	2.687
		Pressure (mPa)	28.021	16.748	4.645	3.330	2.928	11.011	0.422	0.422	3.472	22.817
Aluminium	-	Frequency (Hz)	47.581	78.139	126.933	151.698	158.928	71.936	146.763	146.763	216.522	263.254
		Velocity (mm/s)	7.720	12.655	12.352	15.585	21.335	1.140	3.545	3.545	3.837	4.430
		Pressure (mPa)	47.024	28.116	7.796	5.590	4.916	18.481	0.708	0.708	5.827	38.294
	1 mm	Frequency (Hz)	42.854	70.378	114.324	136.633	143.145	64.679	131.957	131.957	194.682	236.702
		Velocity (mm/s)	6.986	11.340	11.238	13.896	19.045	1.025	3.187	3.187	3.450	3.983
		Pressure (mPa)	38.287	22.423	6.280	4.477	3.899	14.940	0.574	0.574	4.712	30.963
	2 mm	Frequency (Hz)	46.252	75.956	123.387	147.461	154.489	69.895	142.599	142.599	210.380	255.787
		Velocity (mm/s)	7.513	12.285	12.039	15.110	20.691	1.108	3.444	3.444	3.728	4.305
		Pressure (mPa)	44.476	26.451	7.353	5.265	4.619	17.447	0.669	0.669	5.502	36.154
	5 mm	Frequency (Hz)	47.359	77.775	126.342	150.991	158.188	71.598	146.072	146.072	215.503	262.015
		Velocity (mm/s)	7.685	12.594	12.298	15.508	21.230	1.135	3.528	3.528	3.819	4.410
		Pressure (mPa)	46.592	27.842	7.722	5.536	4.867	18.307	0.702	0.702	5.772	37.935
